# Comprehensive characterization of distinct genetic alterations in metastatic breast cancer across various metastatic sites

**DOI:** 10.1038/s41523-021-00303-y

**Published:** 2021-07-16

**Authors:** Soojin Cha, Esak Lee, Hong-Hee Won

**Affiliations:** 1 Samsung Advanced Institute for Health Sciences and Technology (SAIHST), Sungkyunkwan University, Samsung Medical Center, Seoul, Republic of Korea; 2 Meinig School of Biomedical Engineering, Cornell University, Ithaca, NY USA

**Keywords:** Breast cancer, Cancer genomics, Cancer epidemiology

## Abstract

Metastasis is the major cause of death in breast cancer patients. Although previous large-scale analyses have identified frequently altered genes specific to metastatic breast cancer (MBC) compared with those in primary breast cancer (PBC), metastatic site-specific altered genes in MBC remain largely uncharacterized. Moreover, large-scale analyses are required owing to the low expected frequency of such alterations, likely caused by tumor heterogeneity and late dissemination of breast cancer. To clarify MBC-specific genetic alterations, we integrated publicly available clinical and mutation data of 261 genes, including MBC drivers, from 4268 MBC and 5217 PBC patients from eight different cohorts. We performed meta-analyses and logistic regression analyses to identify MBC-enriched genetic alterations relative to those in PBC across 15 different metastatic site sets. We identified 11 genes that were more frequently altered in MBC samples from pan-metastatic sites, including four genes (*SMARCA4*, *TSC2*, *ATRX*, and *AURKA*) which were not identified previously. *ARID2* mutations were enriched in treatment-naïve de novo and post-treatment MBC samples, compared with that in treatment-naïve PBC samples. In metastatic site-specific analyses, associations of *ESR1* with liver metastasis and *RICTOR* with bone metastasis were significant, regardless of intrinsic subtypes. Among the 15 metastatic site sets, *ESR1* mutations were enriched in the liver and depleted in the lymph nodes, whereas *TP53* mutations showed an opposite trend. Seven potential MBC driver mutations showed similar preferential enrichment in specific metastatic sites. This large-scale study identified new MBC genetic alterations according to various metastatic sites and highlights their potential role in breast cancer organotropism.

## Introduction

Breast cancer is the most common type of cancer in women and the second-most common cause of death due to its metastasis or progression to advanced disease^1^. Metastasis is a complex process consisting of cancer cell dissemination, intravasation, circulation in the bloodstream, extravasation, and colonization, with various cell types involved in the tumor and affected organ microenvironment. Numerous studies have identified driver mutations in primary cancer, revealing that the accumulation of driver mutations in cancer cells may contribute to cancer cell proliferation and survival^2^. Several large-scale genomic studies have suggested candidate driver genes of metastatic breast cancer (MBC) using similar methods implemented in studies of primary breast cancer (PBC)^3^. As a result, 31 candidate driver genes of MBC were identified through analyses of various datasets, including those based on whole-genome sequencing, whole-exome sequencing, or targeted gene sequencing with appropriate statistical methods^4–6^. Most of these studies attempted to identify the driver genes enriched in MBC by comparing the frequency of driver genes between unpaired MBC and PBC samples. Among the 31 candidate driver genes of MBC, several (e.g., *ESR1*, *TP53*, and *NF1*) were revealed to be enriched in MBC compared with those in PBC. Moreover, Razavi et al.^7^ identified more than 20 additional MBC-enriched altered genes that were not identified in other studies by comparing the frequency of 468 cancer-related genes between MBC and PBC unpaired samples. The identified MBC-enriched altered genes may have functional impacts on MBC clones in the metastatic sites of breast cancer, which should be elucidated in detail.

Despite efforts to identify the driver genes of MBC and MBC-enriched altered genes compared with those in PBC, further large-scale genomic studies of MBC are needed for several reasons. First, power analyses showed that ~1000 PBC samples were needed to achieve 90% statistical power to identify PBC drivers of low frequency (0.02) at one background mutation per mega base pairs, suggesting that more MBC samples would be required to uncover MBC driver genes with similar power^2,8^. Second, the metastasis of breast cancer may follow a late dissemination model, in which the tumor cells disseminate from a primary tumor site in the late phase of tumorigenesis; therefore, most genetic alterations are likely to be shared between primary and metastatic tumors^7,9,10^. This similarity may make it difficult to identify driver genes that are specifically altered in MBC but rarely detected in PBC, unless matched MBC and PBC samples are compared. Third, intra-genetic heterogeneity of metastatic tumors results in a low allele frequency of candidate driver mutations among the analyzed samples. Compared with gene-level analysis for identifying driver genes, which uses the collective frequencies of multiple variants in each gene, identifying driver mutations by single variant analysis is more challenging because of the expected rarity of each variant and the requirement of a sufficient sample size to ensure appropriate statistical power. Although several driver mutations enriched in MBC were identified by comparing the genomes of patients with MBC and PBC, the number of identified mutations remains low, partly because of the relatively limited scale of previous studies^10^.

Moreover, few studies have investigated the genetic alterations in MBC samples according to their metastatic sites. MBC cells were shown to preferentially metastasize to specific organ sites, in a process referred to as organotropism, including the bone (occurrence rate of 47–60%), liver (19–20%), lung (16–34%), and brain (10–16%), suggesting that different altered genes may contribute to the survival of cancer cells at various metastatic sites^11–13^. Although some molecular characteristics of MBC cells in such metastatic sites have been elucidated, large-scale genomic analyses are required to uncover significantly altered genes in specific metastatic sites.

Therefore, to comprehensively characterize genetic alterations in MBC across various metastatic sites, in this study, we integrated sequencing data of 4268 MBC and 5217 PBC samples from eight different cohorts [Dana-Farber Cancer Institute (DFCI), MD Anderson Cancer Center (MDA), Memorial Sloan Kettering Cancer Center (MSK), Vanderbilt-Ingram Cancer Center (VICC), Foundation Medicine Adult Cancer Clinical Dataset (FMAD), Wellcome Trust Sanger Institute (WTSI), INSERM, and The Cancer Genome Atlas (TCGA)], and analyzed 261 cancer-related genes, including most of the previously identified MBC driver genes, to identify genetic alterations in MBC according to their metastatic sites. We also investigated the possible driver mutations of MBC across different metastatic sites.

## Results

### Pan-metastasis analysis

**Fig. 1:**
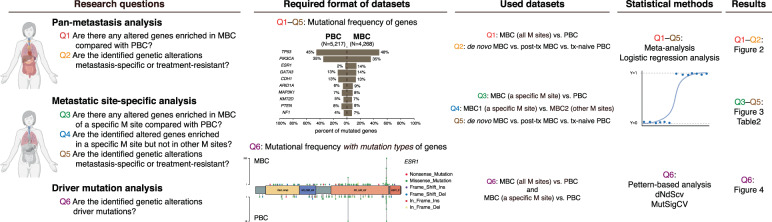
The overall scheme of our study. This study aims to investigate the six research questions using three analyses including pan-metastasis analysis, metastatic site-specific analysis, and driver mutation analysis. For each question, different datasets and data formats were used. Illustrations are created with Biorender.com and maftools^47^.

**Fig. 2:**
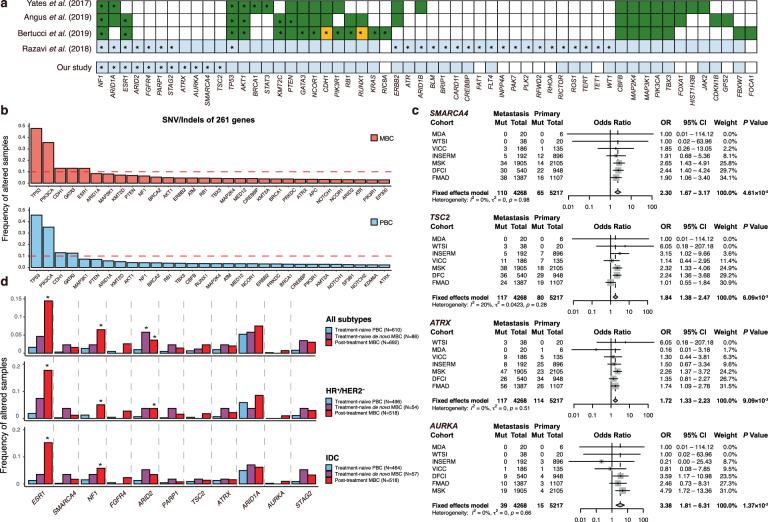
Frequently altered genes in pan-metastatic breast cancer. **a** Candidate MBC driver genes and MBC-enriched altered genes compared with those in PBC are shown. Thirty-one genes identified as MBC driver genes in three previous studies are shown in dark green. These studies used MutSigCV or dNdScv for different datasets to identify MBC driver genes. To identify MBC-enriched altered genes, these studies compared the mutational frequency of the detected driver genes between unpaired MBC and PBC, whereas we and Razavi et al.^7^ compared cancer-related targeted genes between unpaired MBC and PBC. The targeted genes in the study by Razavi et al.^7^ and our study are shown in light blue, and the rest of the targeted genes are listed in Supplementary Table 6 and are described in Razavi et al.^7^, respectively. Asterisks indicate that the tested genes were significantly enriched in MBC for each study (significantly enriched genes in PBC are colored in orange and denoted by an asterisk). To compare the mutational frequency per gene between MBC and PBC, we used meta-analysis and logistic regression analysis, whereas the other studies used Fisher’s exact test. **b** Top 30 frequently altered genes are presented. The upper panel shows MBC samples, and the lower panel shows PBC samples. **c** Forest plots present four significantly altered genes (*SMARCA4*, *TSC2*, *ATRX*, and *AURKA*) that were identified by our meta-analyses and logistic regression analyses and were not shown as MBC-enriched genes in previous studies^7,10,14,15^. Results of fixed-effects meta-analyses and heterogeneity tests between cohorts are shown. *P* values were adjusted by the false discovery rate (FDR). **d** Frequency of samples with mutations of the identified 11 genes according to treatment history and subtypes. Statistically significant difference of the mutational frequency in PBC with de novo MBC or post-treatment MBC are indicated by an asterisk (FDR < 0.05 from logistic regression analysis).

To investigate MBC-specific genetic alterations related to organotropism, we performed three analyses using different datasets and methods (Fig. 1). After performing quality control (QC) and integrating data (Supplementary Tables 1–7 and Supplementary Figs. 1–9), the final number of MBC and PBC samples analyzed was 4268 and 5217, respectively, across seven datasets from the eight cohorts (Table 1). Most of the samples were unpaired between metastasis and primary tumors, except for 17 paired samples from the WTSI cohort. To identify the genes enriched in MBC, we first selected the 261 genes that were concurrently targeted by at least 7 of 11 targeted sequencing panels used in our cohorts (Supplementary Tables 3 and 6). These genes included most of the 31 candidate MBC driver genes identified in three previous studies (Fig. 2a, dark green)^10,14,15^. In particular, the selected genes included 19 of 20 genes identified by at least two studies, suggesting that these 261 genes were adequate for further analyses to identify MBC-specific genetic alterations. Most of the mutated genes showed a long-tail distribution with a low frequency; over 98% of the 261 genes showed a cumulative mutational frequency of <10% for both MBC and PBC samples (Fig. 2b). Ten of the most frequently altered genes in MBC samples exhibited no difference in the frequency of genetic alterations, compared to that in PBC samples, except for *ESR1*, *ARID1A*, and *NF1* which were more frequently altered in MBC samples (Supplementary Fig. 10). This indicates a heterogeneous driver landscape and the importance of aggregated data to identify frequently mutated genes in MBC compared with those in PBC^9,10^.

**Table 1:** Cohort summary.

		DFCI	MDA	MSK	VICC	FMAD	WTSI	INSERM^a^	Total
*N* samples (SNVs/Indels)(metastasis/primary)		540/948	20/6	1905/2105	186/135	1387/1107	38/20	192/896	4268/5217
*N* samples (CNAs)		279/459	NA	1379/1211	139/106	NA	8/7	NA	1805/1783
Receptor status	HR$+$/HER2$-$	NA	NA	572/608	NA	NA	19/10	129/NA	720/618
	HR$+$/HER2$+$	NA	NA	98/49	NA	NA	3/1	12^b^/NA	113/50
	HR$-$/HER2$+$	NA	NA	33/21	NA	NA	6/1	12^b^/NA	51/22
	Triple-negative	NA	NA	75/85	NA	NA	8/7	44/NA	127/92
	NA	540/948	20/6	1127/1342	186/135	1387/1107	2/1	7/896	3269/4435
Histology	Ductal	172/659	0/0	925/1633	138/94	332/804	26/13	NA/658	1593/3861
	Lobular	32/110	1/0	210/256	7/13	49/81	2/0	NA/148	301/608
	Others	336/179	19/6	770/216	41/28	1002/219	9/5	NA/90	2177/743
	NA	0	0	0	0	4/3	1/2	192/0	197/5
Age (mean, years)		57/54	56/47	50/55	56/54	54/52	52/56	NA/59	54/55
Metastatic site^c^	Local recurrence	47	4	13	30	0	25	0	119
	Distant metastases	0	16	765	34	1,382	13	118	2,328
	NA	493	0	0	122	5	0	74	694
Available data	SNVs/Indels	O	O	O	O	O	O	O	–
	CNAs	O	X	O	O	X	O	X	–
Primary cancer samples from		DFCI	MDA	MSK	VICC	FMAD	WTSI	TCGA	–

*SNVs* single-nucleotide variants, *Indel* insertion/deletion, *CNAs* copy number alterations, *NA* not available.

^a^Metastatic samples were from INSERM and primary samples were from TCGA.

^b^Samples with HER2 status of the INSERM cohort were annotated as “HER2 positive”; thus, these samples were included in both the HR+/HER2+ and HR–/HER2+ subgroups.

^c^Samples classified into local recurrence were annotated as “Local recurrence” in the DFCI, MDA, MSK, and VICC cohorts, and were annotated as “Ipsilateral breast/chest wall” or “Regional lymph nodes” and “SYNC_LOCAL_LYMPH_NODE_METASTASIS” in the WTSI cohort.

Next, to identify frequently altered genes in MBC, we conducted meta-analyses to compare the frequency of specific mutation types in each gene between MBC and PBC samples and validated the results by multiple logistic regression analysis adjusted for the cohort. Single-nucleotide variants (SNVs)/insertion and deletions (Indels) in 11 genes were frequently identified in MBC (*ESR1*, *SMARCA4*, *NF1*, *FGFR4*, *ARID2*, *PARP1*, *TSC2*, *ATRX*, *ARID1A*, *AURKA*, and *STAG2* at false discovery rate [FDR] < 5%) compared with those in PBC, and *ESR1* amplifications were frequently identified in MBC samples (Fig. 2a–b and Supplementary Table 8). Among these significantly altered genes, mutation in estrogen receptor 1 (*ESR1*), a known cause of primary hormone therapy resistance, showed the highest odds ratio (OR) (7.39; 95% confidence interval [CI] = 5.90–9.26; FDR = 1.64 × 10^−85^; heterogeneity FDR = 1), consistent with the findings of previous studies (Supplementary Fig. 11)^7,10,14–19^. We identified four MBC-enriched genes, *SMARCA4*, *TSC2*, *ATRX*, and *AURKA* which were not identified in previous large-scale studies for MBC patients (Fig. 2c)^7,10,14,15^.

We compared the mutational frequency of the 11 genes between treatment-naïve PBC, treatment-naïve de novo MBC, and post-treatment MBC samples to examine whether the enriched mutations in MBC were acquired resistant or metastasis-specific using available data from treatment records of patients. Of the 11 genes enriched in MBC, SNV/Indels of *ARID2* were significantly more frequent in treatment-naïve de novo MBC and post-treatment MBC samples than in treatment-naïve PBC samples in all the subtypes and in the IDC subtype (Fig. 2d). Alterations in *ESR1* and *NF1* were significantly frequent only in post-treatment MBC samples, indicating that these might reflect treatment selection. *ESR1* amplification was significantly more frequent in HR + /HER2− and IDC subtype of post-treatment MBC samples, compared to that in treatment-naïve PBC samples (Supplementary Fig. 12).

### Metastatic site-specific analysis

**Fig. 3:**
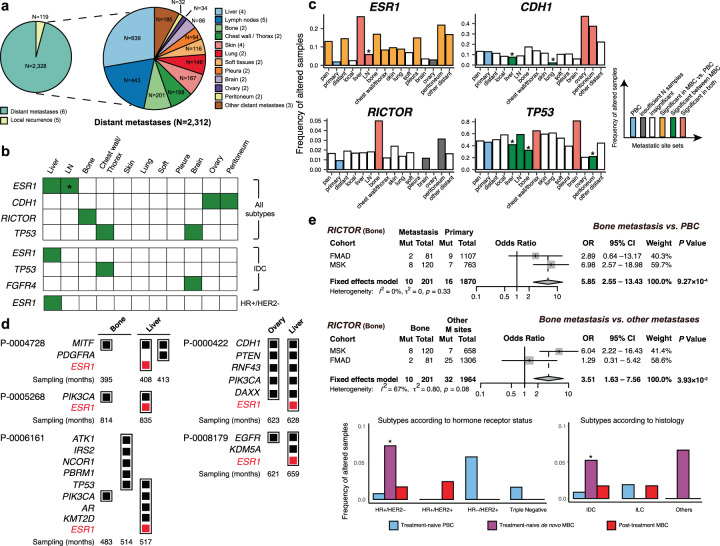
Metastatic site-specific altered genes. **a** Number of samples across 15 metastatic site sets (pan, local, distant, and 12 specific distant sites). In 12 specific metastatic site sets, 16 samples from the MDA cohort were excluded owing to their vague description of metastatic site (e.g., “distant organ metastasis”). The number of cohorts used in each metastatic site set is presented in parentheses. **b** Colored genes indicate those are more frequently altered in the corresponding metastatic site than other sites as well as MBC-enriched compared with PBC (FDR < 5%). Asterisk indicates that mutations in *ESR1* are significantly less frequently altered in the lymph node than other sites while significantly enriched in MBC compared with PBC (FDR < 5%). **c** Distribution of mutational frequency of the four genes (SNVs/Indels) significantly altered in all MBC subtype samples across tissues. Metastatic sites with frequently altered genes compared with both other sites and PBC (FDR < 5%) are shown in red, significant genes compared with other sites in green, significant genes compared with PBC in orange, insignificant genes in white, and genes mutated in only one sample in gray. Asterisks indicate an odds ratio <1. **d** Preference of *ESR1* mutations to the liver is represented by comparing mutations between metastatic liver samples and metastatic bone or metastatic ovarian samples from the same patients. Sampling (months) represents months between sample biopsy for sequencing and diagnosis of PBC. **e** Preference of *RICTOR* mutations to the bone is presented. Comparison of bone metastasis with PBC or other metastatic (M) sites are shown in the forest plot. *P* values were adjusted by FDR. Significantly frequent alterations of *RICTOR* are observed in de novo HR+/HER2− MBC and de novo IDC, as shown in the bottom bar graphs. Asterisks indicate statistical significance at *P* < 0.05.

Considering breast cancer organotropism, there may be distinct genetic alterations in MBC cells at each metastatic site^20,21^. Therefore, we hypothesized that the distinct MBC-enriched altered genes may be associated with particular metastatic sites. To test this hypothesis, we classified the MBC samples into 15 metastatic site sets, including broader categories of pan-metastasis, distant metastasis, and local relapse, and compared the number of samples with altered genes in MBC at specific metastatic site sets with that in PBC using meta-analysis and logistic regression analysis (Supplementary Table 7 and Fig. 3a). These analyses identified 19 genes across 14 metastatic site sets as significantly altered genes in specific metastatic site sets at FDR < 5% (Supplementary Table 8). To investigate the preference of altered genes for specific metastatic sites over other sites, we additionally compared the mutational frequency of 19 genes between MBC samples from metastatic sites using logistic regression analysis (Supplementary Table 9). Of note, four altered genes (*ESR1*, *CDH1*, *RICTOR*, and *TP53*) showed a preference for at least one specific metastatic site in all subtypes of MBC, indicating that these genes tend to metastasize to specific sites rather than other sites as well as they were enriched in MBC compared with PBC (FDR < 5%; Fig. 3b–c and Supplementary Fig. 13). Intriguingly, *ESR1* and *TP53* mutations showed a significant preference for more than one metastatic site in the opposite direction, particularly in the liver and lymph nodes (Table 2). In the liver, *ESR1* mutations were more frequently observed than other sites while *TP53* mutations were less frequently observed than other sites (OR = 3.41 vs. 0.63; 95% CI = 2.67–4.35 vs. 0.52–0.76; FDR = 1.19 × 10^−21^ vs. 4.18 × 10^−5^, respectively). The tendency was opposite in the lymph nodes (OR = 0.31 vs. 1.46; 95% CI = 0.19–0.46 vs. 1.17–1.83; FDR = 2.29 × 10^−6^ vs. 1.67 × 10^−2^ ). Although it should be elucidated in future studies at scale, the observation was consistent with a recent study that showed *TP53* and *ESR1* were the most mutually exclusive gene pair in HR + /HER2− MBC (exclusivity score $\phi =$ 5.4 × 10^−9^)^14^. In the analysis of cases with MBC samples from multiple metastatic sites that were biopsied sequentially, we also found that *ESR1* mutations were more frequently observed in the liver than bone or ovary (Supplementary Table 10 and Fig. 3d).

**Table 2:** Comparison of odds ratios of *ESR1* and *TP53* at various metastatic sites.

M site	Gene	*P*	FDR	OR	95% CI	M site mut	M site wt	Other M sites mut	Other M sites wt
*Liver*	*ESR1*	6.25E-23	1.19E-21	3.41	2.67–4.35	163	420	161	1416
*Lymph nodes*		1.21E-07	2.29E-06	0.31	0.19–0.46	23	361	301	1475
Bone		2.81E-01	1	1.24	0.83–1.82	34	167	290	1669
Chest wall/thorax		1.63E-02	2.93E-01	0.50	0.27–0.85	14	154	310	1682
Skin		1.06E-01	1	0.63	0.35–1.07	15	125	309	1711
Lung		3.80E-02	6.46E-01	0.54	0.29–0.93	13	133	311	1703
Soft		1.15E-02	2.19E-01	0.39	0.17–0.76	8	108	316	1728
Pleura		9.77E-01	1	0.99	0.53–1.72	14	80	310	1756
Brain		6.0E-03	1.08E-01	0.20	0.05–0.53	3	83	321	1753
Ovary		9.13E-02	1	0.18	0.01–0.84	1	33	323	1803
Peritoneum		3.00E-01	1	1.57	0.62–3.47	7	25	317	1811
*Liver*	*TP53*	2.32E-06	4.18E-05	0.63	0.52–0.76	244	339	836	741
*Lymph nodes*		9.27E-04	1.67E-02	1.46	1.17–1.83	225	159	855	921
*Bone*		1.16E-05	2.20E-04	0.49	0.36–0.68	65	136	1015	944
*Chest wall/thorax*		1.49E-05	2.84E-04	2.09	1.50–2.92	109	59	971	1021
Skin		1.79E-02	3.39E-01	1.54	1.08–2.20	87	53	993	1027
Lung		3.93E-03	7.07E-02	1.67	1.18–2.37	89	57	991	1023
Soft		8.91E-01	1	1.03	0.70–1.50	61	55	1019	1025
Pleura		3.52E-02	6.69E-01	0.63	0.41–0.96	37	57	1043	1023
*Brain*		3.94E-08	7.49E-07	4.71	2.78–8.47	70	16	1010	1064
Ovary		4.32E-03	7.78E-02	0.29	0.12–0.65	7	27	1073	1053
*Peritoneum*		1.59E-03	2.86E-02	0.26	0.10–0.57	7	25	1073	1055

Metastatic sites with genes at FDR < 0.05 are shown in italic.

*MBC* metastatic breast cancer, *M site* metastatic site, *M site mut* number of MBC samples from the M site with mutations in the gene, *M site wt* number of MBC samples from the M site with wild-type in the gene, *Other M site mut* number of MBC samples from the other M sites with mutations in the gene, *Other M sites wt* number of MBC samples from the other M sites with wild-type in the gene, *CI* confidence interval, *OR* odds ratio.

To test the effect of intrinsic subtypes of the samples on the associations between the four identified metastatic sites-enriched genes (*ESR1*, *CDH1*, *RICTOR*, and *TP53*) and their metastatic sites (liver, lymph nodes, bone, chest wall/thorax, brain, ovary, and peritoneum), we performed a logistic regression analysis with a covariate of histology or receptor status. The associations of *ESR1* mutations with liver metastasis and *RICTOR* mutations with bone metastasis were significant, irrespective of the intrinsic subtypes. The preference of altered *CDH1* to ovarian and peritoneum metastasis was likely to be related to the preference of ILC subtype for ovary and peritoneum since we observed statistically significant association between ILC subtype and *CDH1* mutations for both ovarian and peritoneum metastases (*P* < 0.01, two-sided Fisher’s exact test), consistent with a previous study^22^. We also found that *RICTOR* mutations were prevalent in the bone and were also frequently observed in de novo treatment-naïve MBC samples, particularly in HR+/HER2− and IDC subtypes (Fig. 3e). These results supported MBC organotropism in which distinct mutations may be involved in metastasis to specific metastatic sites.

### Driver mutation analysis

**Fig. 4:**
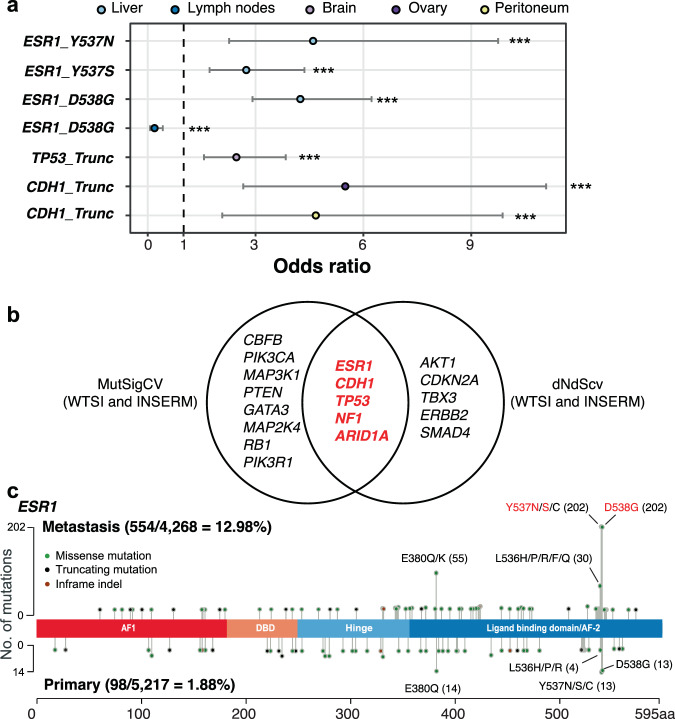
Suggestive driver mutations according to metastatic sites. **a** Significant MBC driver mutations across metastatic sites are shown by comparing 27 candidate driver mutations from MBC of a specific metastatic site with other metastatic sites or PBC. Presented results are from the logistic regression analysis for mutational frequency of 27 candidate driver mutations between MBC samples from different metastatic sites. Truncating mutations are represented by “Trunc” and missense mutations are shown by their amino acid change. Odds ratios and 95% CI for each mutation were shown and asterisks indicate statistical significance at *FDR* < 0.01. **b** MBC driver genes of WTSI and INSERM cohort data which had sufficient background mutations from the results of MutSigCV and dNdScv. **c** Mutation types of *ESR1* shown in a lollipop plot. Red-colored variants are candidate driver mutations enriched in MBC samples. *X*-axis indicates amino acid position.

For the 19 significantly frequently altered genes (FDR < 5%) in MBC (Supplementary Table 8), we assessed the potential functional impact of each variant using computational algorithms and a knowledge-based approach (pattern-based analysis) to identify candidate driver mutations in these genes (Supplementary Fig. 14 and Supplementary Tables 11–12). Twenty-seven mutations across 14 metastatic site sets were suggested as potential driver mutations (Func score $\ge$5), most of which were also suggested as driver mutations with high driver likelihood (>0.5) in a recent study (91%; 20 out of 22 variants analyzed in the study)^23^. Furthermore, seven of the 27 variants were significantly frequently observed in specific metastatic sites compared with other sites and were enriched in MBC compared with those in PBC (FDR < 5%) (Fig. 4a and Supplementary Tables 13–14). Intriguingly, we identified that *ESR1* p.D538G was significantly frequently observed in the liver while less frequent in the lymph nodes than other metastatic sites consistent with our findings from metastatic site-specific analysis at a gene level (Fig. 3) (OR = 4.24 vs. 0.19; 95% CI = 2.91–6.23 vs. 0.07–0.42; FDR = 1.96 × 10^−12^ vs. 7.71 × 10^−3^). We additionally confirmed that our candidate driver mutations of MBC were also detected by other tools for driver analysis (MutSigCV and dNdScv) (Fig. 4b)^24,25^. In breast metastasis, p.Y537 and p.D538 mutations in *ESR1* were related to organotropism among many other hotspot mutations (Fig. 4c). We also found that truncated mutations of *CDH1* were frequently observed in peritoneum metastasis consistent with the results of metastatic site-specific analyses (Figs. 3b and 4a).

## Discussion

We comprehensively identified the frequently altered genes and driver mutations in MBC across 15 metastatic sites using 261 cancer-related genes with rigorous statistical analyses of 4268 MBC and 5217 PBC samples from eight cohorts. In addition to replicating the findings from recent large-scale genomic studies for MBC, we identified MBC-specific SNVs/Indels in *SMACRA4*, *TSC2*, *ATRX*, and *AURKA* which were not identified in previous large-scale studies^7,10,14,15^. We also found preference of mutations in four genes (*ESR1*, *CDH1*, *RICTOR*, and *TP53*) to specific metastatic sites. Furthermore, *ESR1* and *TP53* showed a mutually exclusive tendency of organotropism for the liver and lymph nodes, which should be validated in a large independent dataset (Table 3). Driver mutation analysis also supported our findings of organotropism, particularly for *ESR1* mutations. Thus, our study characterized metastatic site-specific genetic alterations at a large scale and suggested that distinct genetic alterations may be involved in the different metastatic sites of MBC, supporting breast cancer organotropism.

The novel genes frequently altered in MBC compared with PBC have previously been reported to be involved in metastasis-related mechanisms. For example, down-regulation of *SMARCA4* expression inhibited the proliferation, invasiveness, and motility of breast cancer cells in vitro and suppressed metastasis in breast cancer mouse models, suggesting a role of these genes in metastasis^4,5^. In addition, a high expression level of *SMARCA4* or *AURKA* was associated with poor survival or metastasis-free survival in breast cancer patients^26,27^. Furthermore, in pancreatic neuroendocrine tumors, aberrant *ATRX* and *DAXX* expression was associated with lymph node metastasis and distant metastasis by causing the abnormal lengthening of telomeres, and was also associated with shorter disease-free survival and disease-specific survival^28,29^. These results suggest that genes identified in this study may be involved in the general metastatic process of breast cancer.

Stephen Paget studied 735 autopsies of female breast cancer patients and proposed the ‘seed-and-soil’ hypothesis that metastasis does not occur by chance but metastatic tumor cells have a preference for specific organs^30^. To elucidate what genetic factors affect organotropism, it is required to analyze many autopsy samples for multisite metastases, but collecting such samples at scale is challenging^31^. Our approach may be one of the most feasible approaches to investigating breast cancer organotropism, which combines ‘cohort-wise comparison’ of mutational frequency between MBC samples from different metastatic sites as well as between MBC and PBC samples at scale and ‘comparison of multi-metastatic MBC samples’ from the same patients in a relatively small sample size. However, it is important to note that our analyses may be affected by the fact that the site of sampling was the most clinically accessible site, and may not reflect that most patients with MBC have multiple metastatic sites.

Among the 19 altered genes identified by the metastatic site-specific analysis, four genes showed high mutational frequency at specific tissues, further supporting the organotropism of MBC. *RICTOR* was most frequently altered in bone-metastatic breast cancer samples (10/201 = 5%), which is consistent with a previous study showing that bone-derived mesenchymal stem cells (MSCs) with aberrant *RICTOR* expression inhibited breast cancer bone metastasis by repressing osteolytic destruction and cancer-associated fibroblasts^32^. Considering the plasticity of cells between naïve MSCs and breast cancer cells, the higher mutational frequency of *RICTOR* may be related to the bone tropism of MBC cells^33^. Although *TP53* was also frequently mutated in various tissues of our MBC samples, this alteration was most frequently identified in the brain (70/86 = 81%). A previous study also observed frequently mutated *TP53* in 23 central nervous system-metastatic breast cancer patients, supporting that *TP53*-mutant cells may preferentially metastasize to the brain^34^.

There are several limitations to our study. First, we focused on a limited number of genes (261 genes) that were analyzed largely by targeted sequencing. Deep, whole-exome, or whole-genome sequencing in large cohorts is required for unbiased screening of novel genes involved in metastasis and the related mechanisms. Second, we were not able to investigate tumor evolution, but rather could only provide insights into tumor epidemiology, as most of our analyzed samples did not involve primary-metastatic matched pairs like other recent studies^7,14,15^. Therefore, the recurrently altered genes identified by our analysis of largely unmatched metastatic samples may include somatic mutations that might also exist in primary tumors. Direct comparisons of mutational patterns in primary-metastatic matched pairs at scale are necessary to identify and confirm mutations that are specific to MBC. Third, despite the large sample size, the source of the analyzed datasets was heterogeneous in terms of sequencing technologies, sequencing depths, and mutation callers used. To account for this heterogeneity, we analyzed mutations observed only in concurrently targeted sequencing regions in all cohorts and utilized both meta-analysis and regression analysis adjusted for the cohort. Furthermore, treatment history was available only for a small portion of the samples (610 treatment-naïve PBC samples, 86 treatment-naïve de novo MBC samples, and 692 post-treatment MBC samples). Our findings need to be replicated in further studies with a large number of treatment-naïve samples to determine whether the identified mutations were metastasis-specific or acquired resistant. Finally, our suggested driver mutations should be validated experimentally although they were confirmed using three different approaches (pattern-based analysis, MutSigCV, and dNdScv).

In conclusion, we identified distinct genetic alterations of MBC according to specific metastatic sites using large-scale analyses. The molecular characteristics of MBC cells at specific metastatic sites discovered in our study may be considered as biomarkers or therapeutic targets of MBC patients with specific metastases.

## Methods

### Cohort description

We used SNVs, Indels, copy number alterations (CNAs), and clinical data from eight different cohorts (Table 1). Details for each cohort are described below. SNVs/Indels and CNAs data were extracted from the selected samples as described below (Supplementary Figs. 1–2 and Supplementary Tables 1 and 2). Because the MSK, WTSI, INSERM, and TCGA cohorts used matched normal-tumor pairs, whereas the others used a stringent process for filtering germline variants, all mutations were regarded as somatic mutations (Supplementary Table 3).

Data from three cohorts [Dana-Farber Cancer Institute (DFCI), MD Anderson Cancer Center (MDA), and Vanderbilt-Ingram Cancer Center (VICC)] were obtained from AACR Genomics Evidence Neoplasia Information Exchange (GENIE) version 8.0 (https://www.synapse.org/#!Synapse:syn7222066/wiki/405659)^35^. Foundation Medicine Adult Cancer Clinical Dataset (FMAD) data were obtained from dbGAP (phs001179.v1.p1), whereas other data [Wellcome Trust Sanger Institute (WTSI) and INSERM] were obtained from the supplementary data of published studies^10,17,36–38^. Data of the Memorial Sloan Kettering Cancer Center (MSK) cohort were obtained from both a published study by Razavi et al.^7^ and GENIE version 8.0. We used female patient samples, and all data from each cohort contained a set of metastatic samples and mostly comprised unmatched primary samples, except for WTSI cohort samples which consisted of only paired samples. We excluded patients who had multiple samples from multiple metastatic sites to avoid redundant results. The Cancer Genome Atlas (TCGA)-BRCA dataset was used as the primary cancer data for the INSERM cohort. Although the original analysis of the INSERM study used a previous version of the TCGA-BRCA data, we downloaded a mutation annotation format (MAF) file of the latest version (version: gdc-1.0.0, file date: 20170930, number of analyzed samples = 986) of TCGA-BRCA from the Genomic Data Commons data portal (GDC; https://portal.gdc.cancer.gov/) called by MuTect and input to GRCh37 using maf2vcf.pl and vcf2maf.pl (https://github.com/mskcc/vcf2maf) in the GENIE process internally. The details of each cohort dataset are described below.

All data for DFCI, MDA, and VICC cohorts were obtained from GENIE version 8.0^35^. In “data_clinical_sample.txt,” samples were selected when CANCER_TYPE == “Breast Cancer” and SAMPLE_TYPE== “Metastasis” for metastatic breast cancer (MBC) samples and SAMPLE_TYPE == “Primary” for primary breast cancer (PBC) samples. Using sex information from “data_clinical_patient.txt,” male samples were excluded. Samples analyzed by targeted sequencing with panels for all exonic regions of targeted genes, and not hotspot regions, were included (DFCI-ONCOPANEL-1/2/3, MDA-409-V1, VICC-01-T5A, and VICC-01-T7). Histological information was obtained from ONCOTREE_CODE in “data_clinical_sample.txt,” which described invasive ductal carcinoma as “IDC” and invasive lobular carcinoma as “ILC.” Other types of histology were regarded as “Others.” Information regarding metastatic sites was obtained from SAMPLE_TYPE_DETAILED in “data_clinical_sample.txt” when available. Age information was also obtained from “data_clinical_sample.txt” at the sequencing report. The SNVs and insertions/deletions (Indels) data were obtained from selected samples in “data_mutations_extended.txt.” For DFCI and VICC, available copy number alterations (CNAs) data were selected from the selected samples in “data_CNA.txt.” The CNAs values include: low-level gain (1); high-level amplification (2), which was regarded as amplification; as well as deep loss (−2) and single-copy loss (−1), which were regarded as deletion. We selected high-level amplification (2) and deep loss (−2) deletion for harmonization with data from other cohorts and for minimizing false-positives.

Data for the MSK cohort were obtained from both a published study by Razavi et al.^7^ (downloaded from cBio Portal at https://cbioportal.org on Dec 19, 2017) and GENIE version 8.0. In these datasets, 670 MBC and 722 PBC samples overlapped, 111 MBC and 100 PBC samples were only available in the published study (Razavi et al.^7^), and 1190 MBC and 1431 PBC samples were only available in GENIE version 8.0. We combined all available samples from both sources in pan-metastatic analysis, whereas samples from Razavi et al.^7^ were used in other analyses as well, including metastatic site-specific analyses and survival analysis, owing to the availability of the necessary clinical information such as metastatic sites, receptor status, and survival time and status. In the analysis of mutational frequencies of MBC-enriched genes, we also used information of de novo MBC status in data of Razavi et al.^7^ Selected MBC and PBC samples were both annotated as PRIMARY_SITE == “Breast” and annotated as SAMPLE_TYPE == “Metastasis” and SAMPLE_TYPE == “Primary,” respectively. Because the target panels of the MSK cohort were all exonic regions of the targeted genes, all samples analyzed by any target panels of MSK were selected (MSK-IMPACT341/410/468). Histological information was obtained from TUMOR_SAMPLE_HISTOLOGY in “data_clinical_sample.txt.” Samples annotated as “Breast Invasive Ductal Carcinoma” were regarded as “IDC,” “Breast Invasive Lobular Carcinoma” as “ILC,” and others as “Others.” Metastatic site and age-related information were obtained from SAMPLE_SITE and INVASIVE_CARCINOMA_DX_AGE in “data_clinical_sample.txt,” respectively. Information on receptor status, overall survival months, and overall survival status were obtained from OVERALL_RECEPTOR_STATUS_PATIENT, OS_MONTHS, and OS_STATUS in “data_clincal_patient.txt,” respectively. The SNV/Indels data were extracted from selected samples in “data_mutations_extended.txt,” and CNAs data were selected from the selected samples in “data_CNA.txt.” There were three levels of CNA values: high-level amplification (2), which is regarded as amplification, as well as deep loss (−2) and single-copy loss (−1.5), which are regarded as deletions. We selected the CNA values described as high-level amplification (2) and deep loss (−2) for harmonization of these data with other cohorts and for minimizing false-positives. In the mutation data of Razavi et al.^7^, there were different names for the same genes, including *KMT2A*, *KMT2D*, *MLL*, and *MLL2*. We converted the old name of the gene symbols “MLL” and “MLL2” to “KMT2A” and “KMT2D,” respectively. Moreover, we excluded duplicate variants shown in 9 MBC samples and 15 PBC samples, and we excluded variants in CDKN2B-AS1 annotated by Variant Effect Predictor (VEP), CDKN2A p14ARF.

FMAD data were accessed from dbGAP (phs001179.v1.p1), which includes mutation data (SNVs/Indels) in a MAF file and clinical data^36^. As the MAF file was aligned to the GRCh38 reference genome, we converted the genome coordinates to GRCh37 using the liftOver tool (https://genome.ucsc.edu/cgi-bin/hgLiftOver) for further analyses. MBC and PBC samples were selected and annotated as classification_of_tumor == “metastasis” and “primary,” respectively. Samples of FMAD data were analyzed using a cancer gene panel for all exonic regions. Information on histology, age at diagnosis, and metastatic sites were used as described in the clinical data. The SNVs/Indels data were extracted from the selected samples.

Data from the WTSI cohort were obtained from the supplementary materials of a published paper for 386 MBC samples of a relapsed cohort analyzed by targeted sequencing and/or whole-genome sequencing^10^. We used 208 samples with matched normal tissues analyzed by targeted sequencing or both targeted sequencing and whole-genome sequencing for SNVs/Indels analysis, as well as 45 samples analyzed by whole-genome sequencing or both targeted sequencing and whole-genome sequencing for CNA analysis. After excluding samples biopsied from more than one site, we finally selected 53 MBC female samples and 31 PBC female samples that were annotated as SAMPLE_CODE == “DISTANT_METASTASIS,” “LOCAL_RELAPSE” or “SYNC_LN,” and SAMPLE_CODE == “PRIMARY,” respectively. There were 17 metastasis primary paired samples. Information on histology, receptor status, and age was obtained from CODED_HISTO, ER_HER2_PRIMARY, and Age_at_diagnosis, respectively. To determine the receptor status, we evaluated the estrogen receptor as a hormone receptor (HR). Information on metastatic sites was obtained from SAMPLE_CODE. The SNVs/Indels data were obtained from the selected samples as described above, and CNAs data were obtained from the selected samples as described above. Before converting the mutation file to a MAF format file, we corrected Indel variants for harmonization into the MAF format by adjusting the chromosomal start position, end position, reference alleles, and alternative alleles. CNA data were annotated as effect == “AMP” or “HOM_DEL,” and we selected all CNA data because the CNA values included a copy number of more than five as amplification and zero copy number as homo deletion.

Data for the INSERM cohort were acquired from supplementary materials of a published retrospective study of metastatic breast cancer^17^. In the study, as TCGA-BRCA data were used as the primary breast cancer data for the INSERM cohort, we downloaded and analyzed the latest version of TCGA-BRCA for primary cancer of the INSERM cohort as described below. Raw samples were analyzed by whole-exome sequencing of a single metastatic site, and 196 samples that had mutations in 261 genes remained. Information regarding sex was not available. Information regarding the HR status and metastatic sites was used as described in Table 1, and samples annotated as HER2+ were classified into HR+/HER2+ and HR−/HER2−, as our classification considered more specific subtypes. The SNV/Indel data for the selected samples were used. Before converting the mutation file to a MAF format file, we corrected Indel variants for harmonization to the MAF format by adjusting the chromosomal start position, end position, reference alleles, and alternative alleles. As CNAs data were not available for each sample, this cohort was not included in our CNAs analysis.

TCGA-BRCA data were used as the primary cancer for the INSERM cohort. We used the latest version of the TCGA-BRCA data downloaded from the GDC in a MAF file called by MuTect (version: gdc-1.0.0, file date: 2017-09-30, number of analyzed samples = 986).^37,38^ As the file was aligned to reference genome version GRCh38, we converted the genome coordinates to GRCh37 using the liftOver tool (https://genome.ucsc.edu/cgi-bin/hgLiftOver). Clinical data for samples in the MAF file were used in our study, and only samples from female patients were included. Age and histological information were obtained from age_at_diagnosis and primary_diagnosis described in the clinical data, respectively. The SNV/Indel data for all samples were used. As CNAs data for INSERM were not available, we did not use the CNAs data of TCGA.

### Sample selection

For most of the analyses, except for metastatic site-specific analysis with multi-metastatic samples, we used female patient samples (except for the INSERM cohort which did not provide information regarding sex) biopsied from just one site to avoid redundant counts of genetic alterations in multiple samples from the same patients. We selected samples analyzed by whole-exome sequencing or targeted sequencing of all exonic regions for each gene after excluding samples with only hotspot regions sequenced (Supplementary Tables 1–3). Most of the selected samples were unpaired between primary and metastatic cancer, except 17 paired samples from the WTSI cohort. After variant QC, samples with no variants or too many variants were excluded as described in the Supplementary Information (Supplementary Tables 4–5 and Supplementary Figs. 3–9). The final sample numbers were 4268 MBC and 5217 PBC samples for SNVs/Indels data, and 1805 MBC and 1783 PBC samples for CNAs data. If relevant information was available, the samples were classified into subtypes according to their receptor status, histology, or specific metastatic site. Samples from local recurrence sites were composed of those from ipsilateral and contralateral breast relapse, chest wall, or regional lymph nodes, and other samples were regarded as those of distant metastases. For metastatic site-specific analysis with multi-metastatic samples, we used 48 patient samples from at least two metastatic sites (Supplementary Table 10 and Fig. 3d). For the analysis of mutational frequency based on treatment records, we used data of 610 treatment-naïve PBC samples, 86 treatment-naïve de novo MBC samples and 692 post-treatment MBC samples from Razavi et al. which provided information for de novo status of MBC samples and treatment records for the samples^7^.

### Multi-region samples

To validate organotropism of specific genes, we examined 48 multi-metastatic site samples (Supplementary Table 10) which were excluded in other analyses. After processing and QC with the same pipeline as analyses without multi-metastatic site samples, we checked whether four identified MBC-enriched genes for specific metastatic sites (*ESR1*, *CDH1*, *RICTOR*, and *TP53*) were observed in multi-region samples.

### Data integration

Clinical data and SNVs/Indels data were available for all cohorts, whereas CNAs data were available for only four cohorts DFCI, MSK, VICC, and WTSI. Although eight different cohorts were used in our study, downstream analyses were performed on seven datasets of MBC and PBC because the INSERM and TCGA datasets were compared as metastatic samples and primary samples, respectively (Table 1). Because the analytical pipelines of each cohort varied, we harmonized the data formats and obtained a unified annotation of genes and variants. For FMAD and TCGA cohort data, we matched genome coordinates to GRCh37 from GRCh38 using the liftOver tool (https://genome.ucsc.edu/cgi-bin/hgLiftOver). Other data were aligned to a reference of the GRCh37 version in the original files. Then, gene symbols of SNVs/Indels and CNAs data were transformed into the HGNC symbols when gene symbols in the data from each cohort differed from the HGNC symbols according to the latest version (19,198 protein-coding genes; ftp://ftp.ebi.ac.uk/pub/databases/genenames/new/tsv/locus_groups/protein-coding_gene.txt). For SNVs/Indels data, to generate a uniform mutation file annotated by the same version of an annotation tool, we converted mutation files from each cohort into MAF files using publicly available scripts (vcf2maf.pl and maf2vcf.pl) in GENIE data integration (https://github.com/mskcc/vcf2maf) and bcftools (https://samtools.github.io/bcftools). Each variant in the MAF file was annotated by VEP version 90^35^. After converting of gene symbols to HGNC symbols as described above, we unified the CNAs format that contained the same gene symbols and the status of amplification or deletion.

### Quality control of data for SNVs/Indels data

After integrating the datasets, we preprocessed the data by following QC steps for SNVs/Indels data (Supplementary Fig. 1 and Supplementary Table 4). After VEP annotation, we excluded variants with no reference allele, alternative allele, or duplicated variants in the same sample. We discarded the variants for which gene symbols were different before and after VEP annotation.

We also excluded variants with low coverage (<10 depth) or low variant allele frequency (<0.01 VAF) (Supplementary Figs. 3–5). To analyze functionally effective mutations, mutations annotated as “5′ Flank,” “3′ Flank,” “5′ UTR,” “3′ UTR,” “Intron,” or “Silent” were excluded; the final mutation data of SNVs/Indels were composed of variants classified as “Frame_Shift_Del,” “Frame_Shift_Ins,” “In_Frame_Del”, “In_Frame_Ins,” “Missense_Mutation,” “Nonsense_Mutation,” “Nonstop_Mutation,” “Splice_Region,” “Splice_Site,” or “Translation_Start_Site.” Before exclusion of variant classes, silent mutations were not found in most cohorts (Supplementary Fig. 6).

Finally, we excluded variants observed in outlier samples with a large number of mutations (Supplementary Fig. 7). The final numbers of SNVs and Indels were 21,369 and 20,026 for 4268 MBC samples and 5217 PBC samples, respectively.

### QC for CNAs data

After HGNC conversion, we filtered out variants that showed a value of –1 or 1 in the DFCI and VICC cohorts, or a value of –1.5 in the MSK cohort (low CNAs filter) to strictly analyze CNAs generated from targeted sequencing data (Supplementary Figs. 8 and 9). The final numbers of CNAs were 7416 and 5781 for 1805 MBC and 1783 PBC samples, respectively.

### Focusing on 261 genes

As most of the cohorts targeted different sets of genes by various targeted sequencing methods, we limited our analyses to concurrently targeted regions to avoid potential bias. For our analyses, we selected a total of 261 genes that were retained in at least 7 of the 11 (60%) targeted sequencing panels used in six cohorts. To investigate whether the 261 genes included important known and novel genes in MBC and PBC, we listed candidate MBC driver genes as well as candidate PBC driver genes identified by previous large-scale analyses, and compared the previously identified genes with the 261 genes (Supplementary Table 6)^10,14,15,39^. To identify driver genes, Bertucci et al. used MutSigCV for MBC samples analyzed by whole-exome sequencing, whereas Angus et al. and Yates et al. used dNdScv for MBC samples analyzed by whole-genome sequencing and targeted sequencing, respectively^10,14,15^. Nik-Zainal et al.^39^ also used dNdScv for PBC samples analyzed by WGS. We regarded the 261 genes as a representative gene set to investigate MBC driver genes, as they included most of the MBC candidate driver genes and PBC candidate driver genes.

### Statistical analysis

To identify frequently altered genes in MBC, inverse variance-weighted meta-analyses were conducted based on the numbers of mutation carriers in MBC and PBC. We performed a separate meta-analysis for three mutation types: SNVs/Indels, amplifications, and deletions. Joint ORs and 95% CIs were calculated by assuming a fixed-effects model based on the Cochran-Mantel-Haenszel method when the heterogeneity between cohorts was low (*q*-value ≥ 0.05), tested by Tarone’s Q test using *I*^2^ statistics adjusted by the FDR. When the heterogeneity between cohorts was significant (*q*-value < 0.05), joint ORs and 95% CIs were calculated by assuming a random-effects model based on the restricted maximum-likelihood (REML) method. Joint *P* values were adjusted by the FDR, and meta-analyses were conducted using the “metafor” and “meta” packages in R (version 3.5.1).

To validate the results of the meta-analysis for pan-metastases, metastatic site-specific analysis and driver mutation analysis for comparing MBC with PBC, and multivariable logistic regression analyses were conducted with metastasis status as a dependent variable and mutation carrier status as an independent variable after adjustment for cohorts. For the analysis of comparing mutational frequency between MBC samples from different metastatic sites, we compared the mutational frequency of MBC samples from a specific metastatic site with other metastatic sites for 19 MBC-enriched genes (FDR < 5%) using only MSK(R) and FMAD cohort samples (N = 2,160) by multivariable logistic regression analysis with the metastatic site as a dependent variable after adjustment for cohorts. ORs and 95% CIs were estimated, and *P* values were adjusted by the FDR. We presented organotropism-related genes when they were statistically significant from both analyses (comparing mutational frequency between MBC samples from different metastatic sites and between MBC and PBC samples).

Because our data were composed of eight different cohorts, both strategies (heterogeneity test of meta-analyses and multivariable logistic regression analyses with a covariate of cohorts) were used to determine that our results were unlikely to be cohort-specific.

To investigate the effect of treatment status on 11 metastasis-enriched genetic alterations, we conducted multivariable logistic regression analysis between treatment-naïve PBC samples and treatment-naïve *de novo* MBC samples or post-treatment MBC samples for all subtypes and specific subtypes according to receptor status or histology.

### Analysis of driver mutations in MBC

To investigate whether identified MBC-enriched genes across metastatic sites were driver mutations, we performed two strategies as follows considering data type (targeted sequencing data or WES data). First, we performed pattern-based analysis for all datasets. To investigate the functional effects of the frequently altered genes in MBC at the variant level, we applied the following heuristic strategies to identify candidate driver mutations using the mutational pattern of cancer genes that oncogenes (OGs) tend to be recurrently altered in hotspot regions, whereas tumor suppressor genes (TSGs) tend to be truncated by gaining truncating mutations (nonsense, splice-site, or frameshift insertions/deletions) (Supplementary Fig. 14)^40,41^. First, the identified MBC-enriched altered genes according to metastatic site sets were classified as OGs, TSGs, or both as annotated in the 723 cancer gene list of Cancer Gene Census (CGC) version 90 of Catalogue of Somatic Mutations in Cancer (COSMIC)^42^. Genes not annotated in the CGC were discarded. Second, for genes in the CGC, we defined a mutational pattern for each gene. For OGs, we counted the number of MBC samples with mutations in hotspot regions defined by Cancer Hotspots (https://www.cancerhotspots.org)^43,44^. For TSGs, we counted the number of MBC samples with truncating mutations (nonsense, splice-site, or frameshift Indels). For genes classified as both OGs and TSGs, we considered the mutational patterns of both OG and TSG for the genes by counting samples with hotspot mutations for OGs and those with truncating mutations for TSGs. Third, to consider the functional impacts of the mutations, we scored each mutation by annotation from three functional annotation tools (Mutation Assessor, PolyPhen2, and SIFT) for OGs and by experimental evidence in the literature that supported tumor suppressor function or increased invasiveness/metastatic potential for TSGs. We included mutations with a high functional impact (score $\ge$5) observed in more than two carriers in meta-analysis and logistic regression analysis. Finally, for the mutations that satisfied the defined mutational pattern of TSG or OG (*N* = 27), which were referred to as candidate driver mutations in our study, the numbers of mutation carriers and non-carriers of MBC were compared with those of PBC per gene by Cochran-Mantel-Haenszel inverse variance-weighted meta-analysis. The REML method was used when the heterogeneity between cohorts was significant. Joint *P* values were adjusted by the false discovery rate for the tested mutation patterns (Supplementary Tables 11–14). Additionally, we compared mutational frequencies of the 27 candidate driver mutations between MBC samples from different metastatic sites by logistic regression analysis adjusted for cohorts to identify MBC-enriched driver mutations preferentially for specific metastatic sites. *P* values were adjusted by FDR.

In addition to pattern-based analysis, to confirm driver mutations identified by pattern-based analysis, we performed additional driver mutation analyses using MutSigCV and dNdScv for WTSI and INSERM MBC datasets which had sufficient background mutations (synonymous mutations)^24,25^.

### Reporting summary

Further information on research design is available in the Nature Research Reporting Summary linked to this article.

## Supplementary information

Reporting Summary

Supplementary information

## Data Availability

The data generated and analyzed during this study are described in the following data record: 10.6084/m9.figshare.14798541^45^. For DFCI, MDA, MSK(A), and VICC cohorts, the data that support the findings of this study are available in the Synapse repository’s Project GENIE archive, version 8.0 under accession syn7222066 (10.7303/syn22228642)^35^. For the FMAD cohort, the data are available in the dbGAP repository under accession https://identifiers.org/dbgap:phs001179.v1.p1^46^. For the TCGA cohort, the data are available in the Genomic Data Commons data portal (GDC; https://portal.gdc.cancer.gov/) called by MuTect. For other cohorts (MSK(R), WTSI, and INSERM), the data are available in supplementary materials of published studies^7,10,17^.

## Code availability

Meta-analysis and logistic regression analysis were performed using standard R (v.3.5.1) from the ‘metafor’ package and ‘glm’ function, respectively. For preprocessing, we used public available pipelines (liftOver, maf2vcf.pl, vcf2maf.pl, bcftools).
