# Supplementary material for: Comprehensive characterization of distinct genetic alterations in metastatic breast cancer across various metastatic sites
Source: NPJ Breast Cancer. 2021 Jul 16;7:93. doi: 10.1038/s41523-021-00303-y (PMC8285498; doi:10.1038/s41523-021-00303-y)
Supplement: Supplementary file 1 — Reporting Summary [file 41523_2021_303_MOESM1_ESM.pdf]

nature research

Corresponding author(s): Anant Madabhushi

Last updated by author(s): Jun 16, 2021

# Reporting Summary

Nature Research wishes to improve the reproducibility of the work that we publish. This form provides structure for consistency and transparency in reporting. For further information on Nature Research policies, see our Editorial Policies and the Editorial Policy Checklist.

## Statistics

For all statistical analyses, confirm that the following items are present in the figure legend, table legend, main text, or Methods section.

| n/a | Confirmed | |
|---|---|---|
| ☐ | ☒ | The exact sample size (*n*) for each experimental group/condition, given as a discrete number and unit of measurement |
| ☐ | ☒ | A statement on whether measurements were taken from distinct samples or whether the same sample was measured repeatedly |
| ☐ | ☒ | The statistical test(s) used AND whether they are one- or two-sided<br>*Only common tests should be described solely by name; describe more complex techniques in the Methods section.* |
| ☐ | ☒ | A description of all covariates tested |
| ☐ | ☒ | A description of any assumptions or corrections, such as tests of normality and adjustment for multiple comparisons |
| ☐ | ☒ | A full description of the statistical parameters including central tendency (e.g. means) or other basic estimates (e.g. regression coefficient) AND variation (e.g. standard deviation) or associated estimates of uncertainty (e.g. confidence intervals) |
| ☐ | ☒ | For null hypothesis testing, the test statistic (e.g. *F*, *t*, *r*) with confidence intervals, effect sizes, degrees of freedom and *P* value noted<br>*Give P values as exact values whenever suitable.* |
| ☐ | ☒ | For Bayesian analysis, information on the choice of priors and Markov chain Monte Carlo settings |
| ☐ | ☒ | For hierarchical and complex designs, identification of the appropriate level for tests and full reporting of outcomes |
| ☐ | ☒ | Estimates of effect sizes (e.g. Cohen's *d*, Pearson's *r*), indicating how they were calculated |

*Our web collection on statistics for biologists contains articles on many of the points above.*

## Software and code

Policy information about availability of computer code

| | |
|---|---|
| Data collection | RTCGAToolbox (Bioconductor 3.12) was used to collect the clinical information corresponding to TCGA BRCA cohort |
| Data analysis | matlab 2019b, python2.7 |

For manuscripts utilizing custom algorithms or software that are central to the research but not yet described in published literature, software must be made available to editors and reviewers. We strongly encourage code deposition in a community repository (e.g. GitHub). See the Nature Research guidelines for submitting code & software for further information.

## Data

Policy information about availability of data

All manuscripts must include a data availability statement. This statement should provide the following information, where applicable:

- Accession codes, unique identifiers, or web links for publicly available datasets
- A list of figures that have associated raw data
- A description of any restrictions on data availability

The data generated and analysed during this study are described in the following data record: https://doi.org/10.6084/m9.figshare.14755374. The majority of the data underlying the claims of this article are openly available in the ten files included in the data record. The remaining data are in the following two files: 'pred_risk_label_survival_data(Sv).xlsx', 'clincopathological_survival_data(Sv).xlsx'. These two files are housed on institutional storage and are not publicly available in order to protect patient privacy as informed consent to share participant-level data was not obtained prior to or during data collection. Requests for access to these data should be directed to the corresponding author.

# Field-specific reporting

Please select the one below that is the best fit for your research. If you are not sure, read the appropriate sections before making your selection.

☒ Life sciences ☐ Behavioural & social sciences ☐ Ecological, evolutionary & environmental sciences

For a reference copy of the document with all sections, see nature.com/documents/nr-reporting-summary-flat.pdf

# Life sciences study design

All studies must disclose on these points even when the disclosure is negative.

| | |
|---|---|
| Sample size | Two cohorts were included in this study with training set composing 78 patient samples and validation set consisting of 219 patients samples. |
| Data exclusions | For training set, the patients with ER+ breast tumor in TNM stage II or in high risk Lymph node negative (LN-) status were included from TCGA BRCA. The rationale behind this exclusion criterion is to keep the relative consistency between the training and validation set in terms of tumor stage to eliminate the co-effect of the tumor stage on the features aside from the effect from inherent tumor aggressiveness. Other inclusion criteria includes ER+ and reasonable amount of tumor for subsequent image analysis. |
| Replication | We independently validated our constructed prognostic model from training set on the validation set to evaluate the robustness of the built model. |
| Randomization | Our study doesn't involve random allocation of patient samples into different experimental groups. Two cohorts of patient samples were collected respectively from two sources (TCGA BRCA and ECOG 2197) with first cohort being training set and the second cohort being validation set. The covariate was controlled by just including the patients with TNM stage II or in high risk LN- breast tumor to keep the relative consistency between the training and validation set in terms of tumor stage. |
| Blinding | During the image analysis and model construction process, we were blinded to the outcome data for the validation set. |

# Reporting for specific materials, systems and methods

We require information from authors about some types of materials, experimental systems and methods used in many studies. Here, indicate whether each material, system or method listed is relevant to your study. If you are not sure if a list item applies to your research, read the appropriate section before selecting a response.

## Materials & experimental systems

| n/a | Involved in the study | |
|---|---|---|
| ☒ | ☐ | Antibodies |
| ☒ | ☐ | Eukaryotic cell lines |
| ☒ | ☐ | Palaeontology and archaeology |
| ☒ | ☐ | Animals and other organisms |
| ☐ | ☒ | Human research participants |
| ☒ | ☐ | Clinical data |
| ☒ | ☐ | Dual use research of concern |

## Methods

| n/a | Involved in the study | |
|---|---|---|
| ☒ | ☐ | ChIP-seq |
| ☒ | ☐ | Flow cytometry |
| ☒ | ☐ | MRI-based neuroimaging |

## Human research participants

Policy information about studies involving human research participants

| | |
|---|---|
| Population characteristics | All the samples included in this study were female patients with early stage ER+ breast cancer from US. |
| Recruitment | The training set St included 78 patients with the digital slides of Formalin-Fixed Paraffin-Embedded (FFPE) IBC tissue. In order to keep relative consistency with respect to tumor stage with validation set, only the patients with Stage II (Tumor, Node, Metastasis staging system) or high risk (tumor size >=1cm) LN- tumors were recruited into this cohort. All the patients with an DFS event (recurrence or death) meeting the inclusion criteria, matched with a set of censored patients (no DFS event) from TCGA BRCA were used to constitute St.<br>Validation set Sv was used as an independent validation set to evaluate the model performance. The ECOG 2197 trial was a prospective, randomized, clinical trial from 1998 to 2007 that recruited patients with IBC (1 to 3 positive LN / LN- with tumor size >= 1cm) to compare the patient’s outcome under two different chemotherapy regimens. 219 patients with ER+ IBC were selected to comprise Sv after the inclusion criteria applied on the 256 patients in ECOG 2197, for whom we had access to both the corresponding de-identified slide images and relevant clinical information. The access to the ECOG dataset involved 2-year process including a proposal review first through ECOG and subsequently through Cancer Therapy Evaluation Program (CTEP) in National Cancer Institute (NCI). |
| Ethics oversight | The study conformed to HIPAA guidelines was approved by the Institutional Review Board (IRB) at University Hospitals |

Ethics oversight

Cleveland Medical Center. IRB No 02-13-42C. The need for written consent from participants was waived due to the use of de-identified retrospective data.

Note that full information on the approval of the study protocol must also be provided in the manuscript.
