# Supplementary material for: Comprehensive characterization of distinct genetic alterations in metastatic breast cancer across various metastatic sites
Source: NPJ Breast Cancer. 2021 Jul 16;7:93. doi: 10.1038/s41523-021-00303-y (PMC8285498; doi:10.1038/s41523-021-00303-y)
Supplement: Supplementary file 2 — Supplementary information [file 41523_2021_303_MOESM2_ESM.pdf]

Supplementary information

Supplementary Figures

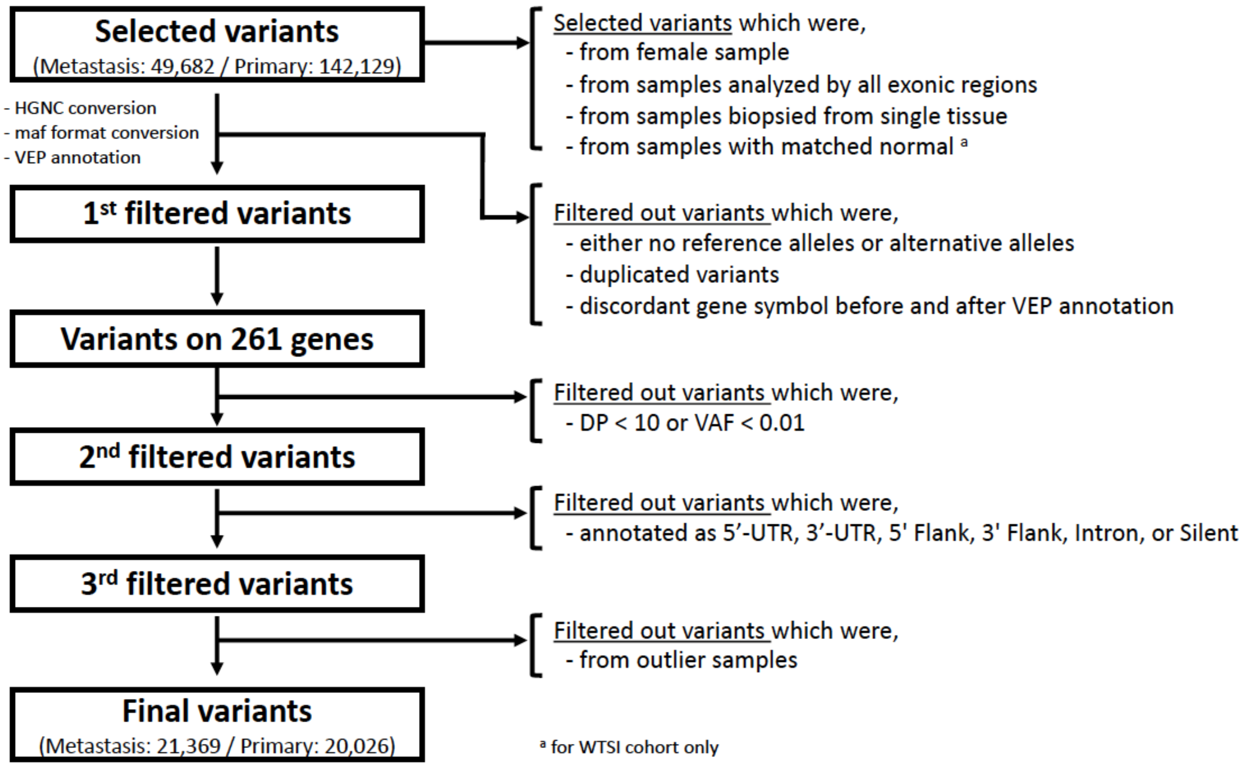

Supplementary Figure 1. Flowchart of quality control for SNVs/Indels.

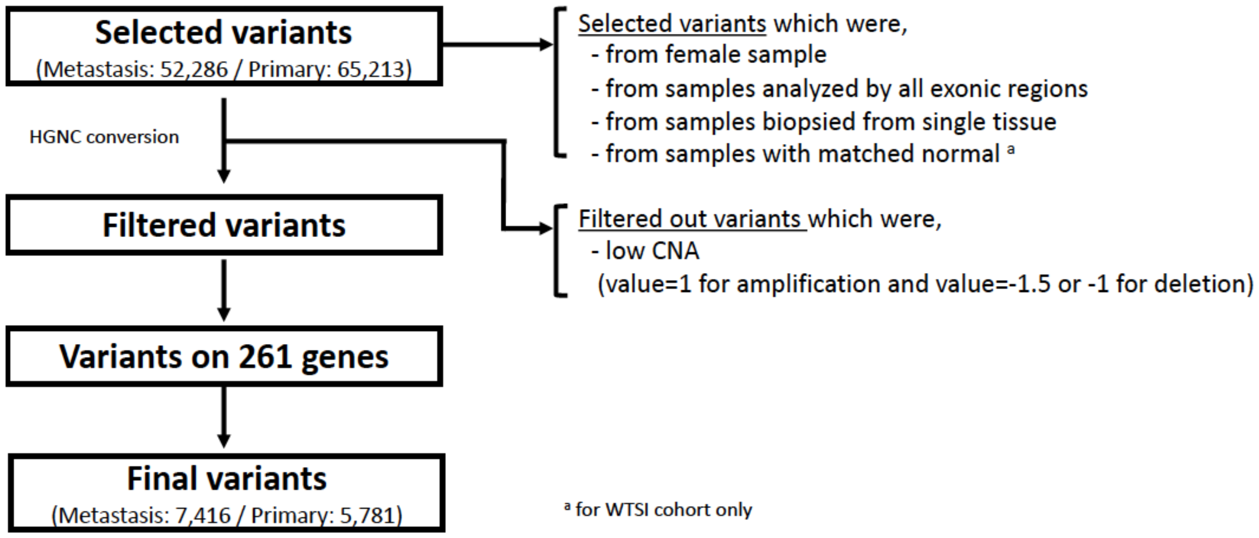

Supplementary Figure 2. Flowchart of quality control for copy number alterations (CNAs).

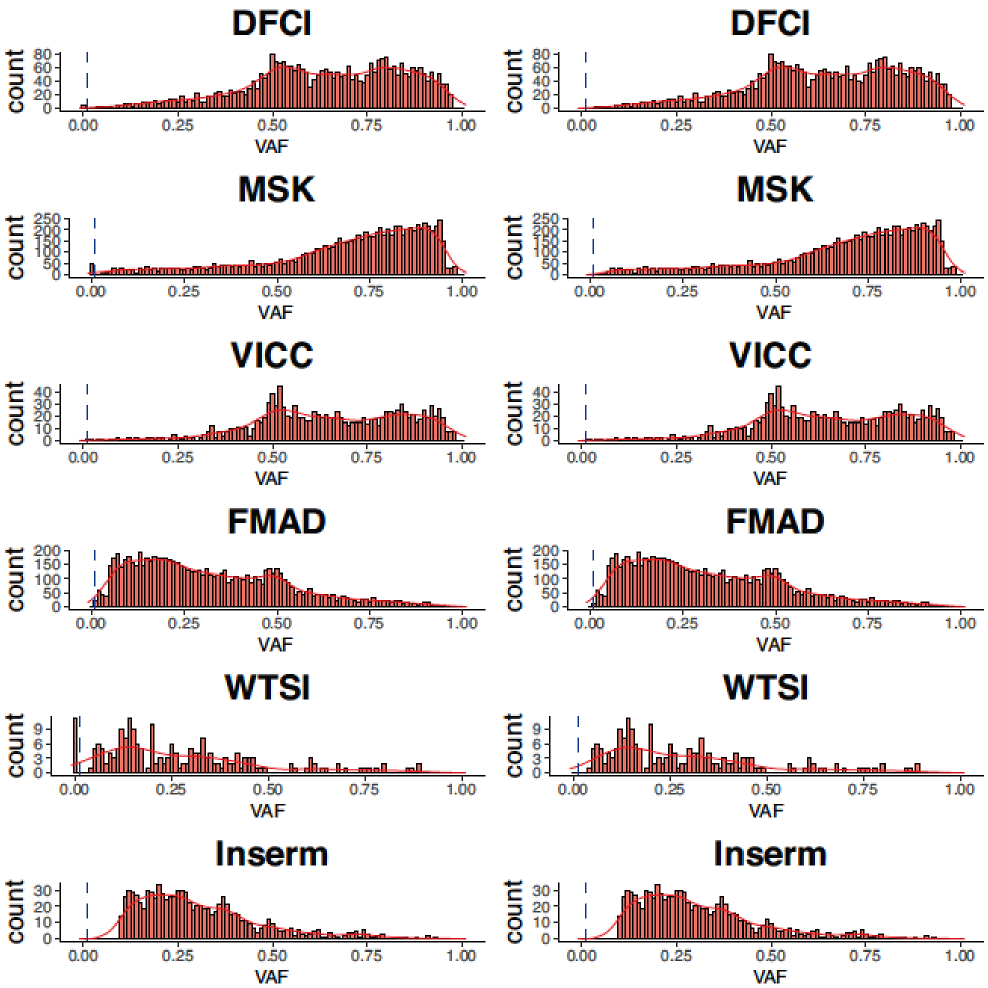

Supplementary Figure 3. Distribution of variant allele frequency (VAF) across cohorts in metastatic breast cancer samples before (left) and after (right) applying depth of coverage and VAF filters.

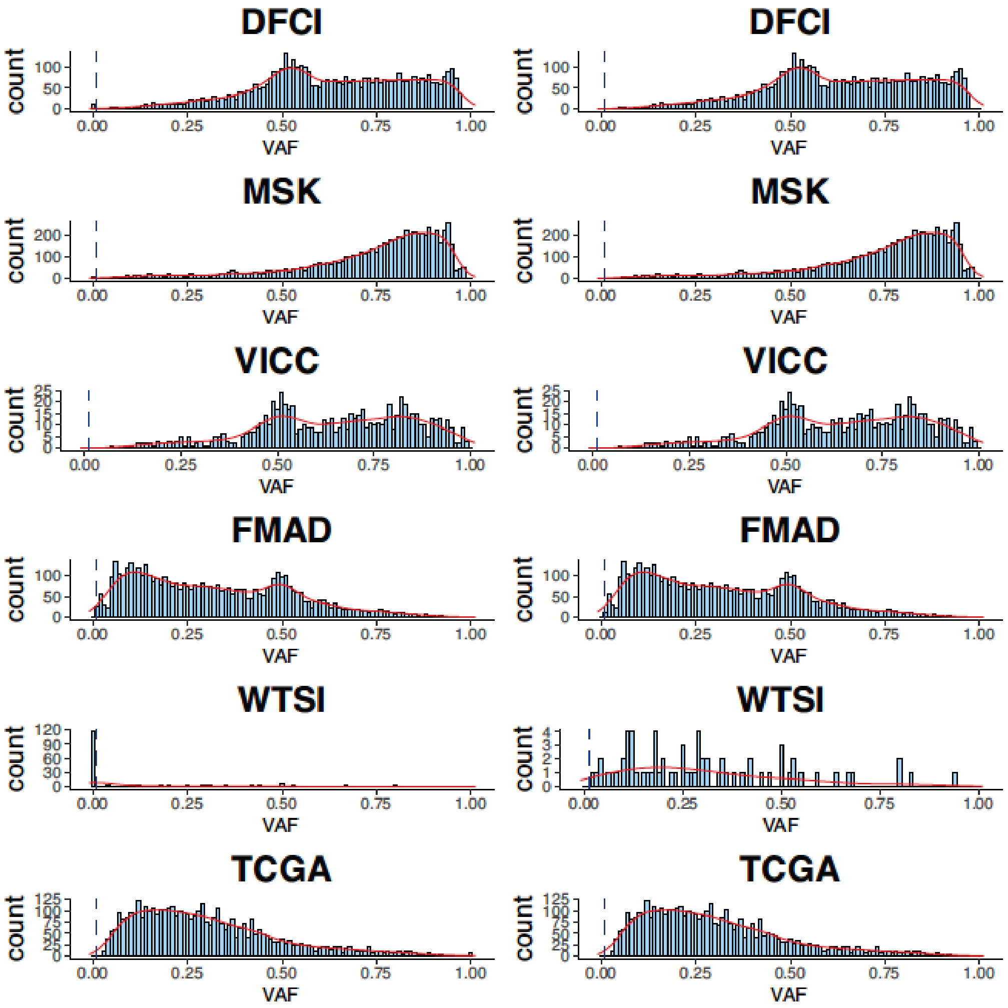

Supplementary Figure 4. Distribution of variant allele frequency (VAF) across cohorts in primary breast cancer samples before (left) and after (right) applying depth of coverage and VAF filters.

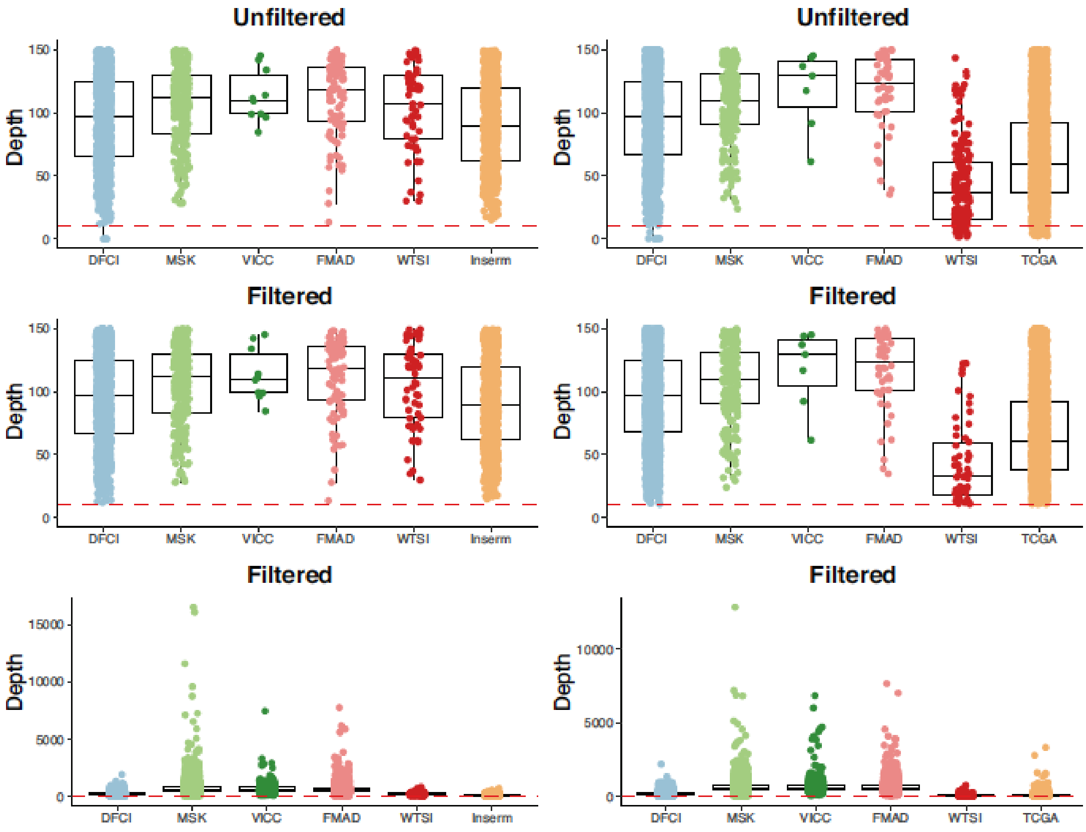

Supplementary Figure 5. Distribution of depth for each variant in samples across cohorts before and after applying depth of coverage and variant allele frequency (VAF) filters. Metastatic breast cancer (MBC) samples and primary breast cancer (PBC) samples are shown in the left and right panels, respectively. Upper and middle panels show the distribution of depth under 150 and bottom panels show the whole range of depth.

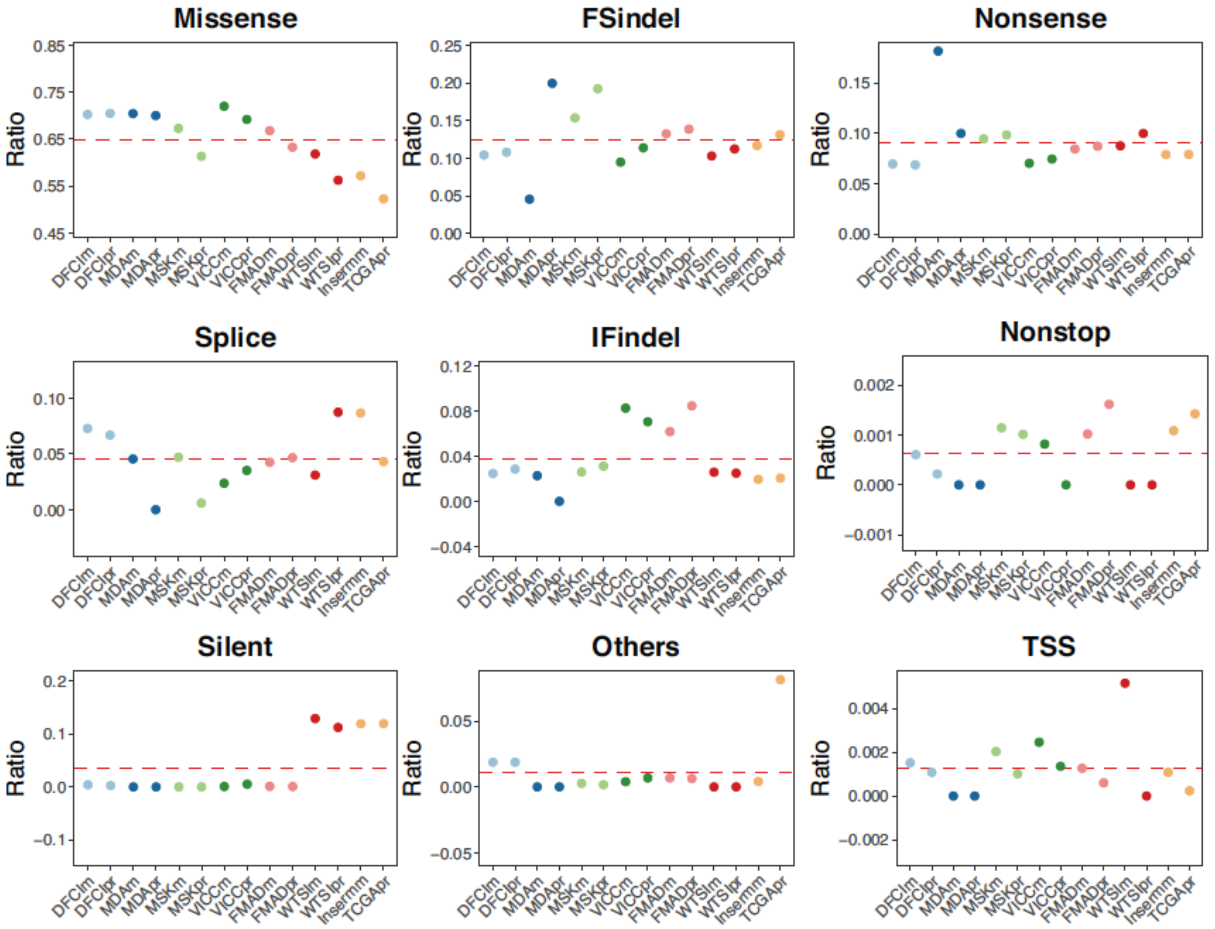

Supplementary Figure 6. Classification of variants in samples across cohorts. Variant classification was annotated by variant effect predictor (VEP). Proportion refers to the number of variants in each category divided by the total number of variants. Mean proportions are shown as red dashed lines. For each cohort, m and pr stand for metastasis and primary, respectively. Variants classified as missense, frameshift insertion/deletion (FSindel), nonsense, splice site or splice region (Splice), in-frame insertion/deletion (IFindel), or nonstop were used in our analyses.

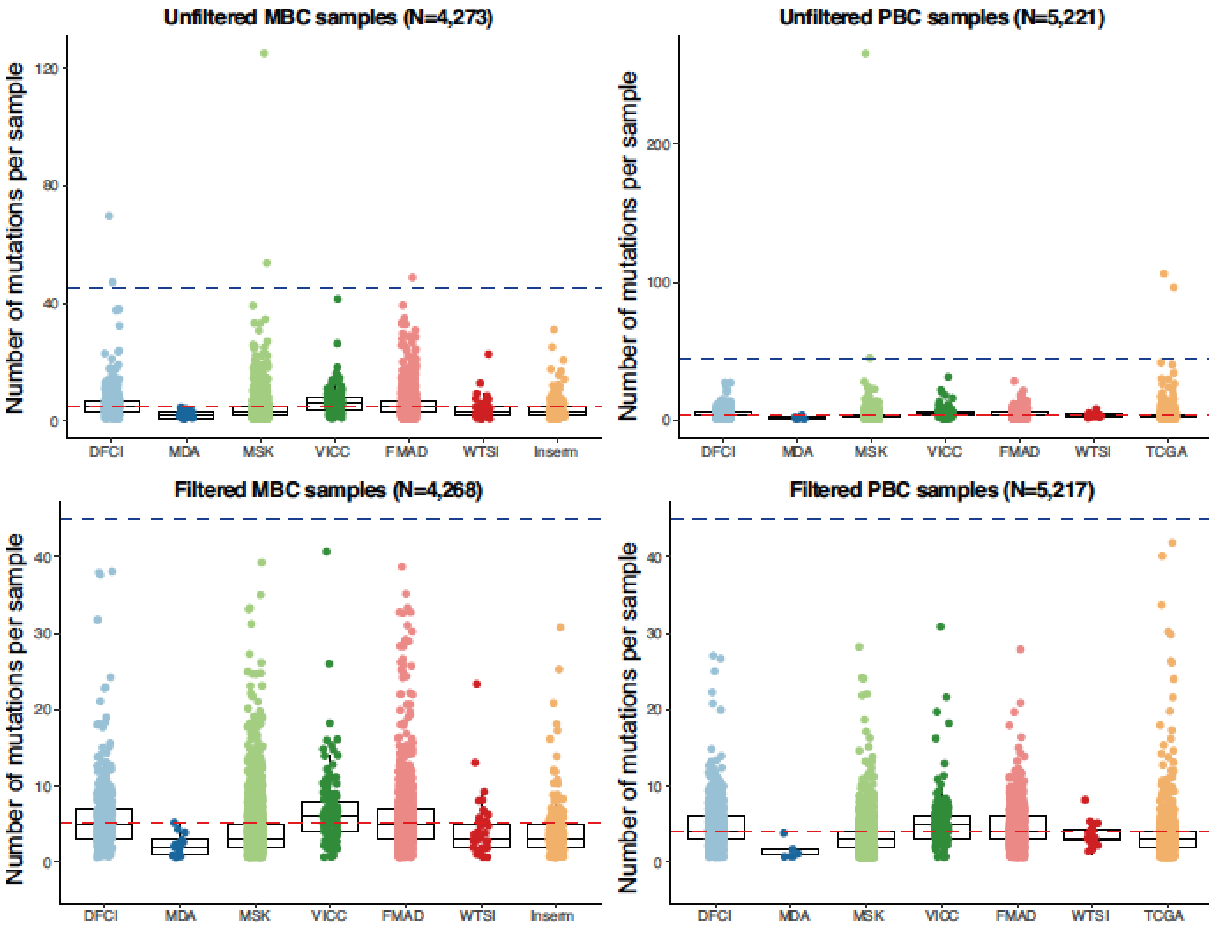

Supplementary Figure 7. Distribution of the number of mutations per sample across cohorts. Number of mutations for 261 genes per sample is shown across cohorts. Metastatic samples are shown in left panels and primary samples are shown in right panels. Samples before applying filters are shown in upper panels and those after filtering are shown in bottom panels. Colors are matched to each analysis set. The blue dotted line represents a threshold of the filter (45) and the red dotted line represents the mean number of mutations per sample for each plot.

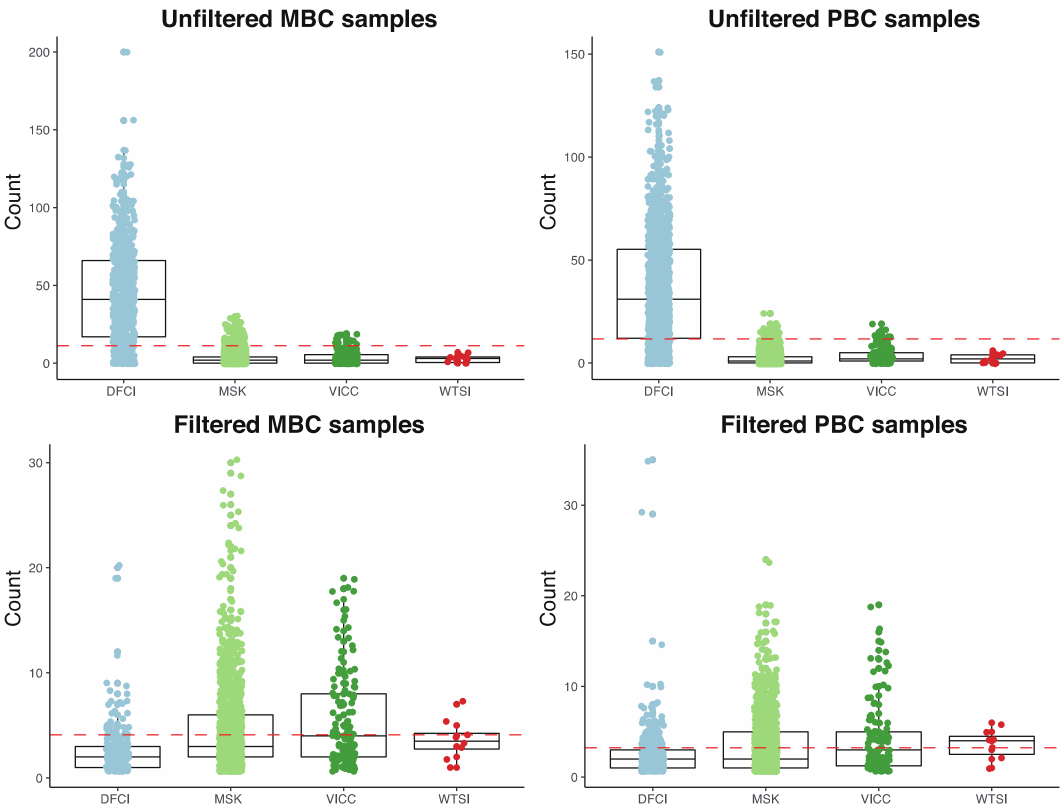

Supplementary Figure 8. Distribution of the number of copy number alterations (CNAs; amplifications and deletions) per sample across cohorts in metastatic breast cancer (MBC) and primary breast cancer (PBC) samples before and after applying the CNAs filter. Number of CNAs for 261 genes per sample is shown across cohorts. Red dotted lines represent the mean number of CNAs per sample of each plot.

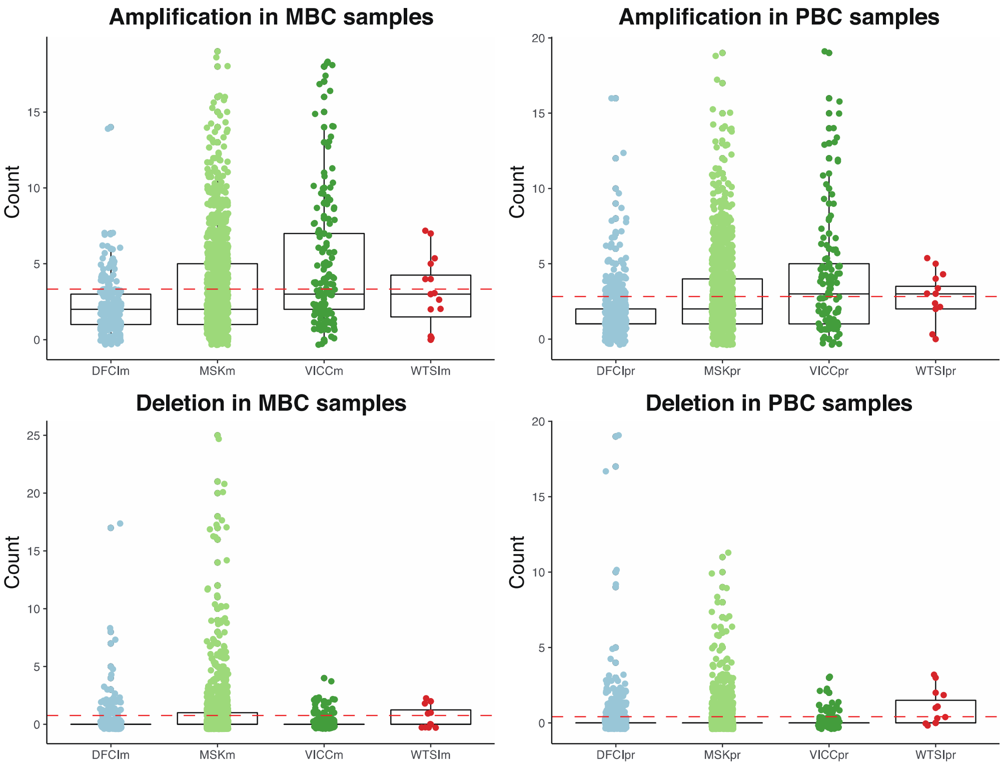

Supplementary Figure 9. Distribution of number of amplifications and deletions per sample across cohorts in metastatic breast cancer (MBC) and primary breast cancer (PBC) samples after quality control.

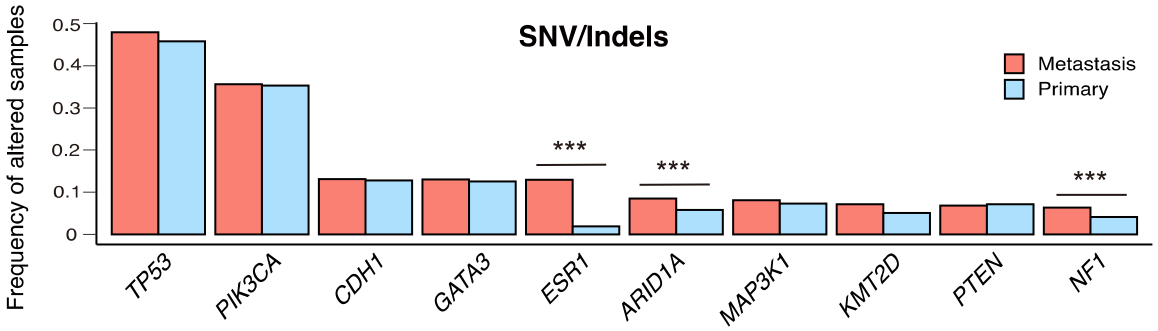

Supplementary Figure 10. Frequency of samples with the 10 most frequently altered genes (related to Fig. 2b). The 10 most frequently altered genes (SNVs/Indels) of metastatic breast cancer (MBC) samples and primary breast cancer (PBC) samples are presented. Asterisks show genes with significantly different mutational frequencies between MBC and PBC based on meta-analysis and logistic regression analysis; ***FDR < 1%.

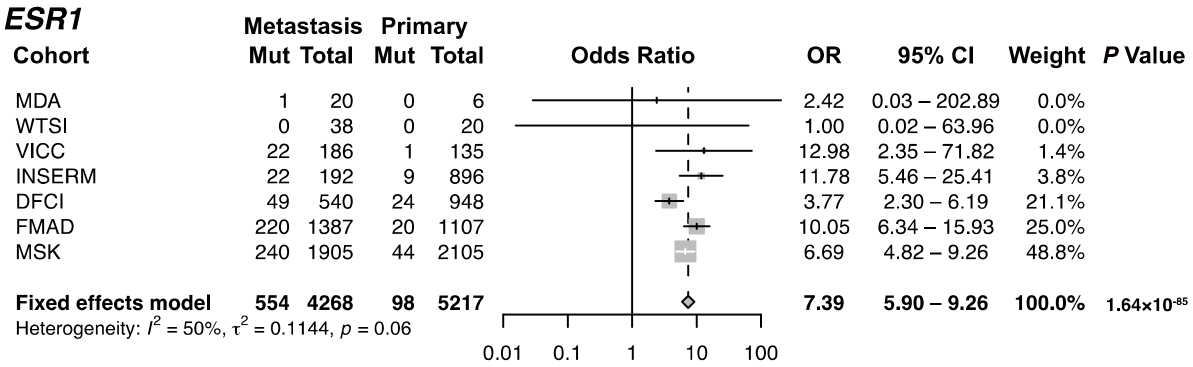

Supplementary Figure 11. Result of meta-analysis for *ESR1* (related to Fig. 2a). *P* values adjusted by false discovery rate are shown.

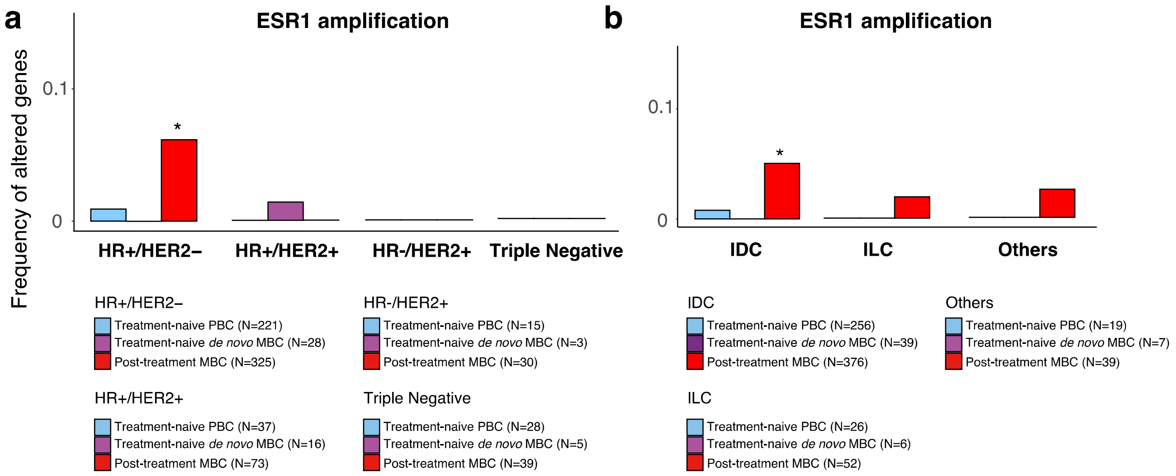

Supplementary Figure 12. Frequency of samples with mutations of 11 genes (single nucleotide variants/insertions and deletions) in treatment-naïve primary breast cancer (PBC), treatment-naïve *de novo* metastatic breast cancer (MBC), and post-treatment MBC (related to Fig. 2d). All samples are from the MSK-Razavi cohort (Cancer Cell.2018.34:427–438) that contains relevant information of treatment history and *de novo* MBC status to classify treatment-naïve PBC, treatment-naïve *de novo* MBC, and post-treatment MBC samples. Results of the *ESR1* amplification for (**a**) four subtypes according to hormone receptor status, and (**b**) histology are presented. Asterisks show statistically significant different mutational frequency of MBC samples compared with PBC samples from logistic regression analysis (FDR < 0.05) after multiple testing correction.

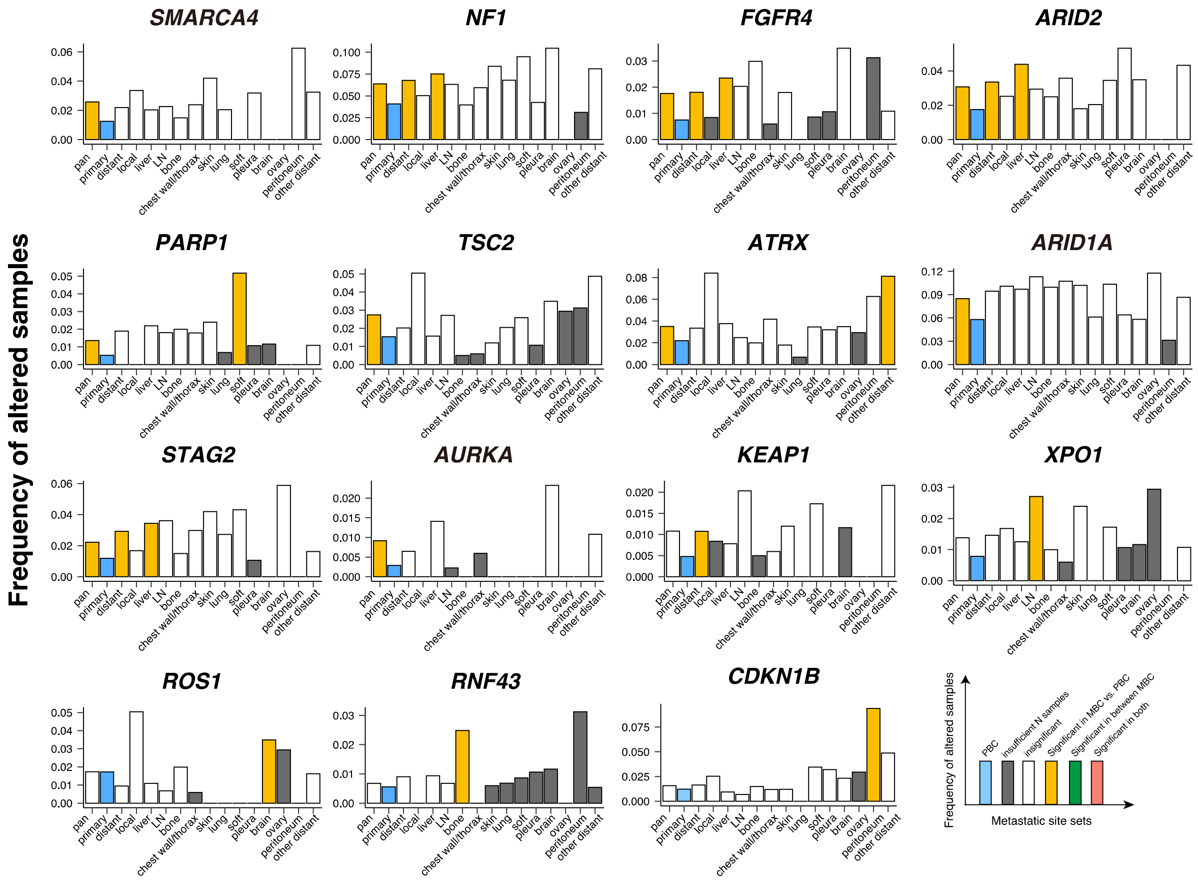

Supplementary Figure 13. Distribution of samples with mutational frequency of 15 metastatic breast cancer (MBC)-enriched genes across tissues (related to Fig. 3b–c). Metastatic sites with significantly frequently altered MBC genes compared with other sites and PBC (FDR < 5%) are shown in red, with significant genes compared with other sites in magenta, with significant genes compared with PBC in yellow, with insignificant genes in white, and with the mutated gene only in one sample are shown in grey.

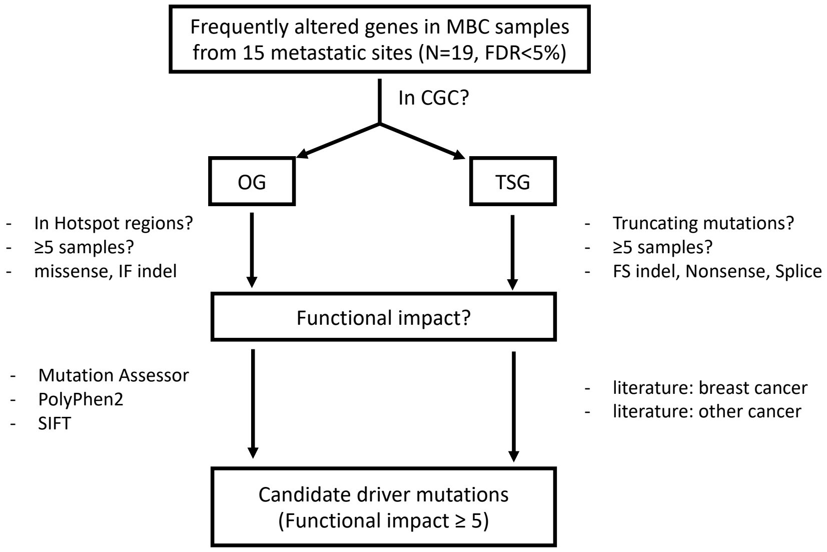

Supplementary Figure 14. Flowchart of identifying driver mutations from metastatic breast cancer (MBC)-enriched altered genes. FDR, false discovery rate; OG, oncogene; TSG, tumor suppressor gene.

Supplementary Tables

Supplementary Table 1. Sample selection for SNVs/Indels data

Sample QC steps	DFCI		MDA		MSK		VICC		FMAD		WTSI		INSERM/TCGA		Total	
	M	P	M	P	M	P	M	P	M	P	M	P	M	P	M	P
**All breast cancer**	693	1,045	327	385	2,743	2,942	205	143	1,415	1,130	236	61	216	1,043	5,835	6,749
**Normal matched**[a]	693	1,045	327	385	2,743	2,942	205	143	1,415	1,130	188	20	216	1,043	5,787	6,708
**Female samples from a single tissue sequenced for all exonic regions**	554	976	26	9	2,008	2,254	187	135	1,402	1,124	42	20	216	974	4,435	5,492
**Samples with at least one mutation in 261 genes**[b]	542	948	20	6	1,907	2,107	186	135	1,388	1,107	38	20	192	898	4,273	5,221
**Non-outliers by mutation numbers**	540	948	20	6	1,905	2,105	186	135	1,387	1,107	38	20	192	896	4,268	5,217
**Final sample numbers**	540	948	20	6	1,905	2,105	186	135	1,387	1,107	38	20	192	896	4,268	5,217

[a] Only available in data from the WTSI cohort.
[b] After variants QC.
Abbreviations: SNVs, single nucleotide variants; Indels, insertions and deletions; M, metastatic breast cancer; P, primary breast cancer; QC, quality control.

Supplementary Table 2. Sample selection for CNAs data

Sample QC steps	DFCI		MSK		VICC		WTSI		Total	
	M	P	M	P	M	P	M	P	M	P
**All breast cancer**	693	1,045	2,743	2,942	205	143	236	61	3,877	4,191
**Normal matched**	693	1,045	2,743	2,942	205	143	31	16	3,672	4,146
**Female samples from a single tissue sequenced for all exonic regions**	554	976	2,007	2,253	187	135	11	11	2,759	3,375
**After CNAs filter**	295	475	1,429	1,273	141	108	8	7	1,873	1,863
**Samples with at least one CNAs in 261 genes**	279	459	1,379	1,211	139	106	8	7	1,805	1,783
**Final sample numbers**	279	459	1,379	1,211	139	106	8	7	1,805	1,783
**Final sample numbers (Amp/Del)**	251/60	422/91	1,312/451	1,148/262	134/30	98/20	6/3	6/3	1,703/544	1,674/376

Abbreviations: CNAs, copy number alterations; M, metastatic breast cancer; P, primary breast cancer; QC, quality control.

Supplementary Table 3. Summary of pipelines of cohorts

Cohort[a]	Panel name	N target genes (exon only)	Tumor cellularity	Depth[b]	Tumor-Normal	Germline variant filtering strategy	SNVs (S), Indels (I) and CNAs (C) caller
DFCI	DFCI-ONCOPANEL-1	302[c]	>20%	222X	Tumor only	1) remove in a panel of normal<br>2) remove MAF ≥ 0.1% in ESP unless also in COSMIC	S: MuTect<br>I: Somatic Indel Detector<br>C: in-house pipeline
	DFCI-ONCOPANEL-2	332[c]					
	DFCI-ONCOPANEL-3	447					
MDA	MDA-409-V1<br>(Ion AmpliSeq Comprehensive Cancer Panel)	409	>20%	>250X	Tumor only	1) remove MAF < 5%<br>2) remove variant coverage < 25<br>3) remove in a paired normal	S/I: TorrentSuite 4.4 - Torrent Variant Caller<br>C: NA
MSK	MSK-IMPACT341	341	>10%	664X	Tumor-Normal	1) remove in a paired normal<br>2) remove MAF ≤ 5% (hotspot ≤ 2%)	S: MuTect<br>I: Pindel and Somatic Indel Detector<br>C: in-house pipeline
	MSK-IMPACT410	410					
	MSK-IMPACT468	468					
VICC	VICC-01-T5A	323	>20%	661X	Tumor only	1) remove MAF < 5% (hotspot < 1%)<br>2) remove variants with strand bias, read location bias, and presence of ≥ 2 normal controls	S/I/C: in-house pipeline
	VICC-01-T7	429					
FMAD	Foundation medicine	287	>20%	644X	Tumor only	1) remove variant in dbSNP142<br>2) remove >2 AC in ExAC (except. known cancer driver germline variant of BRCA1/2 and TP53)<br>3) remove recurrent VUS predicted to be germline assessed by internal algorithm<br>4) remove AF<1% in COSMICv62 and AF<5% not in COMICv62<br>5) retain variant in COSMICv62<br>6) retain all truncations and deletions in known tumor suppressor genes<br>7) optimized in variant of MAF ≥5% for SNVs and ≥10% for indels, and ≥20% for CNA	S/I/C: in-house pipeline
WTSI	WTSI custom	366	>70%	177X	Tumor-Normal	remove variant in a paired normal	S: CaVEMan<br>I: Pindel<br>C: ASCAT
TCGA	WES	all coding genes	NA	107X	Tumor-Normal	remove variant in a paired normal	S: MuTect2<br>I: MuTect2<br>C: SNP array, DNAcopy, Gistic2
INSERM	WES	all coding genes	≥30%	129X	Tumor-Normal	remove variant in a paired normal	S: MuTect<br>I: Scalpel<br>C: ExomeCNV, DNAcopy, Gistic2

[a] Description of pipeline for GENIE data (DFCI, MDA, VICC) was from data_guide_v4.0.pdf and others were from papers for each study.
[b] Mean depth of coverage was estimated in samples used in this study except for MDA which had no available depth information. Mean depth of coverage for MDA was from description in data_guide_v4.0.pdf.
[c] Number of gene list is different from data_guide_v4.0.pdf and released data. We used it from released data.
[d] Abbreviations: MAF, minor allele frequency; ESP, Exome Sequencing Project; COSMIC, Catalogue of Somatic Mutations in Cancer; ExAC, the Exome Aggregation Consortium; SNVs, single nucleotide variants; Indels, insertions and deletions; CNAs, copy number alterations; NA, not available.

Supplementary Table 4. QC for variants in SNVs/Indels data

QC for SNV/Indels[a]	DFCI		MDA		MSK		VICC		FMAD		WTSI		INSERM/TCGA		Total	
	M	P	M	P	M	P	M	P	M	P	M	P	M	P	M	P
**In selected samples**	4,571	6,229	84	15	11,254	8,909	1,525	941	9,015	5,654	284	281	22,949	120,100	49,682	142,129
**1st filtered variants**	4,571	6,229	84	15	11,254	8,909	1,525	941	9,015	5,654	284	281	22,949	120,100	49,682	142,129
**Variants on 261 genes**	3,297	4,637	44	10	8,473	6,927	1,224	737	7,880	4,973	205	196	923	4,217	22,046	23,697
**2nd filtered variants[b]**	3,293	4,625	44	10	8,472	6,927	1,223	737	7,873	4,959	194	80	923	4,217	22,022	21,555
**3rd filtered variants**	3,217	4,525	44	10	8,450	6,914	1,217	728	7,808	4,922	169	71	809	3,369	21,714	20,539
**Final variants**	3,100	4,525	44	10	8,271	6,603	1,217	728	7,759	4,922	169	71	809	3,167	21,369	20,026

[a] Filtering process is shown in Supplementary Fig. 1.
[b] Not available in data from MDA cohort.

Supplementary Table 5. QC for variants in CNAs data

CNAs QC steps[a]	DFCI		MSK		VICC		WTSI		Total	
	M	P	M	P	M	P	M	P	M	P
**In selected samples**	40,322	56,787	10,870	7,719	1,062	679	32	28	52,286	65,213
**Low CNA filter**	999	1,467	10,854	7,714	1,062	679	32	28	12,947	9,888
**In 261 genes**	674	1,060	5,987	4,233	726	463	29	25	7,416	5,781
**Final sample numbers**	674	1,060	5,987	4,233	726	463	29	25	7,416	5,781
**Final sample numbers (Amp/Del)**	553/121	872/188	4,760/1,227	3,707/526	682/44	438/25	24/5	19/6	6,019/1,397	5,036/745

[a] Filtering process is shown in Supplementary Fig. 2.

Supplementary Table 6. 261 gene list used in our study

(a) The 261 gene list

Gene	DFCI-Oncopanel-1	DFCI-Oncopanel-2	DFCI-Oncopanel-3	MDA-409-V1	MSK-IMPACT341	MSK-IMPACT410	MSK-IMPACT468	VICC-01-T5A	VICC-01-T7	FMAD-panel	WTSI-panel
ABL1	Y	Y	Y	Y	Y	Y	Y	Y	Y	Y	Y
AKT1	Y	Y	Y	Y	Y	Y	Y	Y	Y	Y	Y
AKT2	Y	Y	Y	Y	Y	Y	Y	Y	Y	Y	Y
AKT3	Y	Y	Y	Y	Y	Y	Y	Y	Y	Y	Y
ALK	Y	Y	Y	Y	Y	Y	Y	Y	Y	Y	Y
ALOX12B	Y	Y	N	N	Y	Y	Y	Y	Y	Y	N
AMER1	N	N	N	Y	Y	Y	Y	Y	Y	Y	Y
APC	Y	Y	Y	Y	Y	Y	Y	Y	Y	Y	Y
AR	Y	Y	Y	Y	Y	Y	Y	Y	Y	Y	Y
ARAF	Y	Y	Y	N	Y	Y	Y	Y	Y	Y	Y
ARID1A	Y	Y	Y	Y	Y	Y	Y	Y	Y	Y	Y
ARID1B	Y	Y	Y	N	Y	Y	Y	N	Y	N	Y
ARID2	Y	Y	Y	Y	Y	Y	Y	Y	Y	Y	Y
ASXL1	Y	Y	Y	Y	Y	Y	Y	Y	Y	Y	Y
ATM	Y	Y	Y	Y	Y	Y	Y	Y	Y	Y	Y
ATR	N	N	Y	Y	Y	Y	Y	Y	Y	Y	Y
ATRX	Y	Y	Y	Y	Y	Y	Y	Y	Y	Y	Y
AURKA	Y	Y	Y	Y	Y	Y	Y	Y	Y	Y	Y
AURKB	Y	Y	Y	Y	Y	Y	Y	Y	Y	Y	Y
AXL	Y	Y	Y	Y	Y	Y	Y	Y	Y	Y	Y
B2M	Y	Y	Y	N	Y	Y	Y	N	N	N	Y
BAP1	Y	Y	Y	Y	Y	Y	Y	Y	Y	Y	Y
BARD1	N	N	Y	N	Y	Y	Y	Y	Y	Y	N
BCL2	Y	Y	Y	Y	Y	Y	Y	Y	Y	Y	Y
BCL2L1	Y	Y	Y	Y	Y	Y	Y	N	Y	N	Y
BCL6	Y	Y	Y	Y	Y	Y	Y	Y	Y	Y	Y
BCOR	Y	Y	Y	N	Y	Y	Y	Y	Y	Y	Y
BLM	Y	Y	Y	Y	Y	Y	Y	Y	Y	Y	Y
BMPR1A	Y	Y	Y	Y	Y	Y	Y	N	Y	N	N
BRAF	Y	Y	Y	Y	Y	Y	Y	Y	Y	Y	Y
BRCA1	Y	Y	Y	N	Y	Y	Y	Y	Y	Y	Y
BRCA2	Y	Y	Y	N	Y	Y	Y	Y	Y	Y	Y
BRD4	Y	Y	Y	N	Y	Y	Y	N	Y	N	N
BRIP1	Y	Y	Y	Y	Y	Y	Y	Y	Y	Y	Y
BTK	N	N	N	Y	Y	Y	Y	Y	Y	Y	N
CARD11	Y	Y	Y	Y	Y	Y	Y	Y	Y	Y	Y
CASP8	N	N	Y	N	Y	Y	Y	Y	Y	Y	Y
CBFB	N	N	Y	N	Y	Y	Y	Y	Y	Y	Y
CBL	Y	Y	Y	Y	Y	Y	Y	Y	Y	Y	Y
CCND1	Y	Y	Y	Y	Y	Y	Y	Y	Y	Y	Y
CCND2	Y	Y	Y	Y	Y	Y	Y	Y	Y	Y	Y
CCND3	Y	Y	Y	N	Y	Y	Y	Y	Y	Y	Y
CCNE1	Y	Y	Y	Y	Y	Y	Y	Y	Y	Y	Y
CD274	Y	Y	Y	N	Y	Y	Y	N	Y	N	N
CD79B	Y	Y	Y	Y	Y	Y	Y	Y	Y	Y	N
CDC73	Y	Y	Y	Y	Y	Y	Y	Y	Y	Y	Y
CDH1	Y	Y	Y	Y	Y	Y	Y	Y	Y	Y	Y
CDK12	N	N	Y	Y	Y	Y	Y	Y	Y	Y	Y
CDK4	Y	Y	Y	Y	Y	Y	Y	Y	Y	Y	Y
CDK6	Y	Y	Y	Y	Y	Y	Y	Y	Y	Y	Y
CDK8	N	N	Y	Y	Y	Y	Y	Y	Y	Y	Y
CDKN1A	Y	Y	Y	N	Y	Y	Y	N	Y	N	Y
CDKN1B	Y	Y	Y	N	Y	Y	Y	Y	Y	Y	Y
CDKN2A	Y	Y	Y	Y	Y	Y	Y	Y	Y	Y	Y
CDKN2B	Y	Y	Y	Y	Y	Y	Y	Y	Y	Y	Y
CDKN2C	Y	Y	Y	Y	Y	Y	Y	Y	Y	Y	Y
CEBPA	Y	Y	Y	Y	N	Y	Y	Y	Y	Y	Y
CHEK1	N	N	Y	Y	Y	Y	Y	Y	Y	Y	Y
CHEK2	Y	Y	Y	Y	Y	Y	Y	Y	Y	Y	Y
CIC	N	N	Y	Y	Y	Y	Y	Y	Y	Y	Y
CREBBP	Y	Y	Y	Y	Y	Y	Y	Y	Y	Y	Y
CRKL	Y	Y	Y	Y	Y	Y	Y	Y	Y	Y	Y
CRLF2	Y	Y	Y	N	Y	Y	Y	Y	Y	Y	N
CSF1R	N	Y	N	Y	Y	Y	Y	Y	Y	Y	N
CTCF	N	N	Y	N	Y	Y	Y	Y	Y	Y	Y
CTNNB1	Y	Y	Y	Y	Y	Y	Y	Y	Y	Y	Y
DAXX	N	N	Y	Y	Y	Y	Y	Y	Y	Y	Y
DDR2	Y	Y	Y	Y	Y	Y	Y	Y	Y	Y	Y

DICER1	Y	Y	Y	Y	Y	Y	Y	N	Y	N	Y
DIS3	Y	Y	Y	N	Y	Y	Y	Y	Y	Y	N
DNMT3A	Y	Y	Y	Y	Y	Y	Y	Y	Y	Y	Y
EGFR	Y	Y	Y	Y	Y	Y	Y	Y	Y	Y	Y
EP300	Y	Y	Y	Y	Y	Y	Y	Y	Y	Y	Y
EPHA3	Y	Y	N	Y	Y	Y	Y	Y	Y	Y	Y
EPHA5	Y	Y	N	N	Y	Y	Y	Y	Y	Y	N
EPHB1	N	N	N	Y	Y	Y	Y	Y	Y	Y	Y
ERBB2	Y	Y	Y	Y	Y	Y	Y	Y	Y	Y	Y
ERBB3	Y	Y	Y	Y	Y	Y	Y	Y	Y	Y	Y
ERBB4	Y	Y	Y	Y	Y	Y	Y	Y	Y	Y	Y
ERCC2	Y	Y	Y	Y	Y	Y	Y	Y	Y	N	N
ERCC3	Y	Y	Y	Y	Y	Y	Y	N	N	N	N
ERCC4	Y	Y	Y	Y	Y	Y	Y	N	Y	N	N
ERCC5	Y	Y	Y	Y	Y	Y	Y	N	N	N	N
ERG	N	Y	Y	Y	Y	Y	Y	Y	Y	Y	N
ESR1	Y	Y	Y	Y	Y	Y	Y	Y	Y	Y	Y
ETV1	Y	Y	Y	Y	Y	Y	Y	Y	Y	N	Y
ETV6	Y	Y	Y	N	Y	Y	Y	Y	Y	N	N
EZH2	Y	Y	Y	Y	Y	Y	Y	Y	Y	Y	Y
FANCA	Y	Y	Y	Y	Y	Y	Y	Y	Y	Y	Y
FANCC	Y	Y	Y	Y	Y	Y	Y	Y	Y	Y	Y
FANCD2	Y	Y	Y	Y	N	N	N	Y	Y	Y	Y
FANCE	Y	Y	Y	N	N	N	N	Y	Y	Y	Y
FANCF	Y	Y	Y	Y	N	N	N	Y	Y	Y	Y
FANCG	Y	Y	Y	Y	N	N	N	Y	Y	Y	Y
FBXW7	Y	Y	Y	Y	Y	Y	Y	Y	Y	Y	Y
FGFR1	Y	Y	Y	Y	Y	Y	Y	Y	Y	Y	Y
FGFR2	Y	Y	Y	Y	Y	Y	Y	Y	Y	Y	Y
FGFR3	Y	Y	Y	Y	Y	Y	Y	Y	Y	Y	Y
FGFR4	Y	Y	Y	Y	Y	Y	Y	Y	Y	Y	Y
FH	Y	Y	Y	Y	Y	Y	Y	N	Y	N	Y
FLCN	Y	Y	Y	Y	Y	Y	Y	N	Y	N	N
FLT1	Y	Y	Y	Y	Y	Y	Y	Y	Y	Y	Y
FLT3	Y	Y	Y	Y	Y	Y	Y	Y	Y	Y	Y
FLT4	Y	Y	Y	Y	Y	Y	Y	Y	Y	Y	Y
FOXL2	N	N	Y	Y	Y	Y	Y	Y	Y	Y	Y
GATA1	N	N	N	Y	Y	Y	Y	Y	Y	Y	Y
GATA2	N	N	Y	Y	Y	Y	Y	Y	Y	Y	Y
GATA3	Y	Y	Y	Y	Y	Y	Y	Y	Y	Y	Y
GNA11	Y	Y	Y	Y	Y	Y	Y	Y	Y	Y	Y
GNAQ	Y	Y	Y	Y	Y	Y	Y	Y	Y	Y	Y
GNAS	Y	Y	Y	Y	Y	Y	Y	Y	Y	Y	Y
GRIN2A	N	N	N	N	Y	Y	Y	Y	Y	Y	Y
H3F3A	Y	Y	Y	N	N	Y	Y	N	Y	N	Y
HNF1A	Y	Y	Y	Y	Y	Y	Y	N	Y	N	Y
HRAS	Y	Y	Y	Y	Y	Y	Y	Y	Y	Y	Y
IDH1	Y	Y	Y	Y	Y	Y	Y	Y	Y	Y	Y
IDH2	Y	Y	Y	Y	Y	Y	Y	Y	Y	Y	Y
IGF1R	Y	Y	Y	Y	Y	Y	Y	Y	Y	Y	Y
IGF2	N	N	Y	Y	Y	Y	Y	Y	Y	Y	N
IKBKE	N	N	N	Y	Y	Y	Y	Y	Y	Y	Y
IKZF1	Y	Y	Y	Y	Y	Y	Y	Y	Y	Y	N
IL7R	N	N	Y	Y	Y	Y	Y	Y	Y	Y	Y
IRF4	N	N	N	Y	Y	Y	Y	Y	Y	Y	N
IRS2	N	N	N	Y	Y	Y	Y	Y	Y	Y	Y
JAK1	N	N	Y	Y	Y	Y	Y	Y	Y	Y	Y
JAK2	Y	Y	Y	Y	Y	Y	Y	Y	Y	Y	Y
JAK3	Y	Y	Y	Y	Y	Y	Y	Y	Y	Y	Y
JUN	N	N	N	Y	Y	Y	Y	Y	Y	Y	Y
KDM5A	N	N	Y	N	Y	Y	Y	Y	Y	Y	Y
KDM5C	N	Y	Y	Y	Y	Y	Y	Y	Y	Y	Y
KDM6A	Y	Y	Y	Y	Y	Y	Y	Y	Y	Y	Y
KDR	Y	Y	Y	Y	Y	Y	Y	Y	Y	Y	Y
KEAP1	N	Y	Y	Y	Y	Y	Y	Y	Y	Y	Y
KIT	Y	Y	Y	Y	Y	Y	Y	Y	Y	Y	Y
KMT2A	Y	Y	Y	Y	Y	Y	Y	Y	Y	Y	Y
KMT2D	Y	Y	Y	Y	Y	Y	Y	Y	Y	N	Y
KRAS	Y	Y	Y	Y	Y	Y	Y	Y	Y	Y	Y
LMO1	Y	Y	Y	N	Y	Y	Y	Y	Y	Y	N
MAP2K1	Y	Y	Y	Y	Y	Y	Y	Y	Y	Y	Y
MAP2K2	N	N	Y	Y	Y	Y	Y	Y	Y	Y	Y
MAP2K4	Y	Y	Y	Y	Y	Y	Y	Y	Y	Y	Y
MAP3K1	Y	Y	Y	N	Y	Y	Y	Y	Y	Y	Y
MAP3K13	N	N	N	N	Y	Y	Y	Y	Y	Y	Y

MAPK1	Y	Y	Y	Y	Y	Y	Y	N	N	N	Y
MCL1	Y	Y	Y	Y	Y	Y	Y	Y	Y	Y	Y
MDM2	Y	Y	Y	Y	Y	Y	Y	Y	Y	Y	Y
MDM4	Y	Y	Y	Y	Y	Y	Y	Y	Y	Y	Y
MED12	N	N	Y	N	Y	Y	Y	Y	Y	Y	Y
MEF2B	Y	Y	Y	N	Y	Y	Y	Y	Y	Y	N
MEN1	Y	Y	Y	Y	Y	Y	Y	Y	Y	Y	Y
MET	Y	Y	Y	Y	Y	Y	Y	Y	Y	Y	Y
MITF	Y	Y	Y	Y	Y	Y	Y	Y	Y	Y	Y
MLH1	Y	Y	Y	Y	Y	Y	Y	Y	Y	Y	Y
MPL	Y	Y	Y	Y	Y	Y	Y	Y	Y	Y	Y
MRE11	N	N	Y	Y	Y	Y	Y	Y	Y	Y	Y
MSH2	Y	Y	Y	Y	Y	Y	Y	Y	Y	Y	Y
MSH6	Y	Y	Y	Y	Y	Y	Y	Y	Y	Y	Y
MTOR	Y	Y	Y	Y	Y	Y	Y	Y	Y	Y	Y
MUTYH	Y	Y	Y	Y	Y	Y	Y	Y	Y	Y	Y
MYC	Y	Y	Y	Y	Y	Y	Y	Y	Y	Y	Y
MYCL	Y	Y	Y	Y	Y	Y	Y	Y	Y	Y	Y
MYCN	Y	Y	Y	Y	Y	Y	Y	Y	Y	Y	Y
MYD88	Y	Y	Y	Y	Y	Y	Y	Y	Y	Y	Y
NBN	Y	Y	Y	Y	Y	Y	Y	Y	Y	Y	Y
NCOR1	N	N	N	N	Y	Y	Y	Y	Y	Y	Y
NF1	Y	Y	Y	Y	Y	Y	Y	Y	Y	Y	Y
NF2	Y	Y	Y	Y	Y	Y	Y	Y	Y	Y	Y
NFE2L2	Y	Y	Y	Y	Y	Y	Y	Y	Y	Y	Y
NFKBIA	Y	Y	Y	N	N	Y	Y	Y	Y	Y	N
NKX2-1	Y	Y	Y	Y	Y	Y	Y	Y	Y	Y	Y
NOTCH1	Y	Y	Y	Y	Y	Y	Y	Y	Y	Y	Y
NOTCH2	Y	Y	Y	Y	Y	Y	Y	Y	Y	Y	Y
NOTCH3	N	N	Y	N	Y	Y	Y	Y	Y	Y	Y
NOTCH4	N	N	N	Y	Y	Y	Y	Y	Y	Y	Y
NPM1	Y	Y	Y	Y	Y	Y	Y	Y	Y	Y	Y
NRAS	Y	Y	Y	Y	Y	Y	Y	Y	Y	Y	Y
NSD1	N	N	Y	Y	Y	Y	Y	Y	Y	Y	N
NTRK1	Y	Y	Y	Y	Y	Y	Y	Y	Y	Y	Y
NTRK2	Y	Y	Y	N	Y	Y	Y	Y	Y	Y	Y
NTRK3	Y	Y	Y	Y	Y	Y	Y	Y	Y	Y	Y
PALB2	Y	Y	Y	Y	Y	Y	Y	Y	Y	Y	Y
PARP1	N	N	N	Y	Y	Y	Y	Y	Y	Y	N
PAX5	Y	Y	Y	Y	Y	Y	Y	Y	Y	Y	Y
PBRM1	N	Y	Y	Y	Y	Y	Y	Y	Y	Y	Y
PDGFRA	Y	Y	Y	Y	Y	Y	Y	Y	Y	Y	Y
PDGFRB	Y	Y	Y	Y	Y	Y	Y	Y	Y	Y	Y
PHOX2B	Y	Y	Y	Y	Y	Y	Y	N	N	N	N
PIK3C2G	N	N	N	N	Y	Y	Y	Y	Y	Y	Y
PIK3C3	N	N	N	N	Y	Y	Y	Y	Y	Y	Y
PIK3CA	Y	Y	Y	Y	Y	Y	Y	Y	Y	Y	Y
PIK3CG	N	N	N	Y	Y	Y	Y	Y	Y	Y	Y
PIK3R1	Y	Y	Y	Y	Y	Y	Y	Y	Y	Y	Y
PIK3R2	N	N	N	Y	Y	Y	Y	Y	Y	Y	Y
PIM1	Y	Y	Y	Y	Y	Y	Y	N	N	N	Y
PMS1	Y	Y	Y	Y	Y	Y	Y	N	N	N	N
PMS2	Y	Y	Y	Y	Y	Y	Y	Y	Y	Y	N
PNRC1	Y	Y	N	N	Y	Y	Y	Y	Y	Y	N
PPP2R1A	N	N	Y	Y	Y	Y	Y	Y	Y	Y	Y
PRDM1	Y	Y	Y	Y	Y	Y	Y	Y	Y	Y	Y
PRKAR1A	Y	Y	Y	Y	Y	Y	Y	Y	Y	Y	Y
PRKDC	Y	Y	Y	Y	N	N	N	Y	Y	Y	N
PRKN	Y	Y	Y	N	Y	Y	Y	N	Y	N	N
PTCH1	Y	Y	Y	Y	Y	Y	Y	Y	Y	Y	Y
PTEN	Y	Y	Y	Y	Y	Y	Y	Y	Y	Y	Y
PTPN11	Y	Y	Y	Y	Y	Y	Y	Y	Y	Y	Y
RAD50	N	N	Y	Y	Y	Y	Y	Y	Y	Y	Y
RAD51	N	N	Y	N	Y	Y	Y	Y	Y	Y	Y
RAD51C	N	N	Y	N	Y	Y	Y	Y	Y	Y	N
RAD51D	N	N	Y	N	Y	Y	Y	Y	Y	Y	N
RAD52	N	N	Y	N	Y	Y	Y	Y	Y	Y	N
RAF1	Y	Y	Y	Y	Y	Y	Y	Y	Y	Y	Y
RARA	Y	Y	Y	Y	Y	Y	Y	Y	Y	Y	Y
RB1	Y	Y	Y	Y	Y	Y	Y	Y	Y	Y	Y
REL	Y	Y	Y	Y	Y	Y	Y	Y	Y	Y	N
RET	Y	Y	Y	Y	Y	Y	Y	Y	Y	Y	Y
RICTOR	N	N	Y	N	Y	Y	Y	Y	Y	Y	Y
RNF43	N	N	Y	N	Y	Y	Y	Y	Y	Y	Y
ROS1	Y	Y	Y	Y	Y	Y	Y	Y	Y	N	Y

RPTOR	N	N	Y	N	Y	Y	Y	Y	Y	Y	Y
RUNX1	Y	Y	Y	Y	Y	Y	Y	Y	Y	Y	Y
SDHA	N	Y	Y	Y	Y	Y	Y	N	Y	N	N
SDHB	Y	Y	Y	Y	Y	Y	Y	N	Y	N	N
SDHC	Y	Y	Y	Y	Y	Y	Y	N	Y	N	N
SDHD	Y	Y	Y	Y	Y	Y	Y	N	Y	N	N
SETD2	Y	Y	Y	Y	Y	Y	Y	Y	Y	Y	Y
SF3B1	Y	Y	Y	Y	Y	Y	Y	Y	Y	Y	Y
SH2B3	Y	Y	Y	N	N	Y	Y	Y	Y	Y	N
SMAD2	Y	Y	Y	Y	Y	Y	Y	Y	Y	Y	Y
SMAD4	Y	Y	Y	Y	Y	Y	Y	Y	Y	Y	Y
SMARCA4	Y	Y	Y	Y	Y	Y	Y	Y	Y	Y	Y
SMARCB1	Y	Y	Y	Y	Y	Y	Y	Y	Y	Y	Y
SMO	Y	Y	Y	Y	Y	Y	Y	Y	Y	Y	Y
SOCS1	Y	Y	Y	Y	Y	Y	Y	Y	Y	Y	Y
SOX2	Y	Y	Y	Y	Y	Y	Y	Y	Y	Y	Y
SOX9	Y	Y	Y	N	Y	Y	Y	N	Y	N	Y
SPOP	N	N	Y	N	Y	Y	Y	Y	Y	Y	Y
SRC	Y	Y	N	Y	Y	Y	Y	Y	Y	Y	Y
STAG2	Y	Y	Y	N	Y	Y	Y	Y	Y	Y	Y
STAT3	Y	Y	Y	N	N	Y	Y	N	Y	N	Y
STK11	Y	Y	Y	Y	Y	Y	Y	Y	Y	Y	Y
SUFU	Y	Y	Y	Y	Y	Y	Y	Y	Y	Y	Y
SYK	Y	Y	N	Y	Y	Y	Y	Y	Y	Y	N
TBX3	N	N	N	N	Y	Y	Y	Y	Y	Y	Y
TCF7L2	Y	Y	Y	Y	N	Y	Y	N	N	N	Y
TENT5C	Y	Y	Y	N	Y	Y	Y	Y	Y	Y	N
TERT	Y	Y	Y	N	Y	Y	Y	N	N	N	Y
TET2	Y	Y	Y	Y	Y	Y	Y	Y	Y	Y	Y
TGFBR2	N	N	N	Y	Y	Y	Y	Y	Y	Y	Y
TMPRSS2	N	Y	Y	N	Y	Y	Y	Y	Y	N	N
TNFAIP3	Y	Y	Y	Y	Y	Y	Y	Y	Y	Y	Y
TNFRSF14	N	N	N	Y	Y	Y	Y	Y	Y	Y	N
TOP1	N	N	N	Y	Y	Y	Y	Y	Y	Y	Y
TP53	Y	Y	Y	Y	Y	Y	Y	Y	Y	Y	Y
TSC1	Y	Y	Y	Y	Y	Y	Y	Y	Y	Y	Y
TSC2	Y	Y	Y	Y	Y	Y	Y	Y	Y	Y	Y
TSHR	N	N	Y	Y	Y	Y	Y	Y	Y	Y	Y
U2AF1	Y	Y	Y	N	Y	Y	Y	N	Y	N	Y
VHL	Y	Y	Y	Y	Y	Y	Y	Y	Y	Y	Y
WT1	Y	Y	Y	Y	Y	Y	Y	Y	Y	Y	Y
XPO1	Y	Y	Y	Y	Y	Y	Y	Y	Y	Y	Y
ZNF217	Y	Y	Y	N	N	N	N	Y	Y	Y	Y

(b) Comparison to candidate driver genes of MBC and PBC identified in other studies

	MBC			PBC	
**Gene**	**Bertucci et al. (2019) N=617**	**Angus et al. (2019) N=442**	**Yates et al. (2017) N=227**	**Nik-Zainal et al. (2016) N=560**	**In 261 genes?**
TP53	candidate driver	candidate driver	candidate driver	candidate driver	Y
PIK3CA	candidate driver	candidate driver	candidate driver	candidate driver	Y
GATA3	candidate driver	candidate driver	candidate driver	candidate driver	Y
ESR1	candidate driver	candidate driver	passenger	candidate driver	Y
KMT2C	candidate driver	candidate driver	passenger	candidate driver	Y
CDH1	candidate driver	candidate driver	candidate driver	candidate driver	Y
PTEN	candidate driver	candidate driver	candidate driver	candidate driver	Y
NF1	candidate driver	candidate driver	candidate driver	candidate driver	Y
MAP3K1	candidate driver	candidate driver	candidate driver	candidate driver	Y
NCOR1	candidate driver	candidate driver	candidate driver	candidate driver	Y
AKT1	candidate driver	candidate driver	candidate driver	candidate driver	Y
FOCA1	candidate driver	passenger	passenger	passenger	N
KRAS	candidate driver	passenger	passenger	candidate driver	Y
RB1	candidate driver	candidate driver	passenger	candidate driver	Y
TBX3	candidate driver	candidate driver	candidate driver	candidate driver	Y
RIC8A	candidate driver	passenger	passenger	passenger	N
RUNX1	candidate driver	candidate driver	passenger	candidate driver	Y
FBXW7	candidate driver	passenger	passenger	candidate driver	Y
MAP2K4	candidate driver	candidate driver	candidate driver	candidate driver	Y
CBFB	candidate driver	candidate driver	candidate driver	candidate driver	Y
ARID1A	passenger	candidate driver	candidate driver	candidate driver	Y
CDKN1B	passenger	candidate driver	passenger	candidate driver	Y
ERBB2	passenger	candidate driver	candidate driver	candidate driver	Y
FOXA1	passenger	candidate driver	candidate driver	candidate driver	N
GPS2	passenger	candidate driver	passenger	passenger	N
PIK3R1	candidate driver	passenger	candidate driver	candidate driver	Y
BRCA1	passenger	passenger	candidate driver	candidate driver	Y
HIST1H3B	passenger	passenger	candidate driver	passenger	N
JAK2	passenger	passenger	candidate driver	passenger	Y
STAT3	passenger	passenger	candidate driver	passenger	Y
ARID1B	passenger	passenger	candidate driver	candidate driver	Y
KMT2D	passenger	passenger	passenger	candidate driver	Y
AXIN1	passenger	passenger	passenger	candidate driver	N
ATR	passenger	passenger	passenger	candidate driver	Y
MDM2	passenger	passenger	passenger	candidate driver	Y
MLLT4	passenger	passenger	passenger	candidate driver	N
CIC	passenger	passenger	passenger	candidate driver	Y
APC	passenger	passenger	passenger	candidate driver	Y
ATRX	passenger	passenger	passenger	candidate driver	Y
PALB2	passenger	passenger	passenger	candidate driver	Y
ZNF217	passenger	passenger	passenger	candidate driver	Y
AKT2	passenger	passenger	passenger	candidate driver	Y
ASXL1	passenger	passenger	passenger	candidate driver	Y
ATM	passenger	passenger	passenger	candidate driver	Y
BCOR	passenger	passenger	passenger	candidate driver	Y
BRAF	passenger	passenger	passenger	candidate driver	Y
BRCA2	passenger	passenger	passenger	candidate driver	Y
BUB1B	passenger	passenger	passenger	candidate driver	N
CASP8	passenger	passenger	passenger	candidate driver	Y
CBLB	passenger	passenger	passenger	candidate driver	N
CCND1	passenger	passenger	passenger	candidate driver	Y
CCND3	passenger	passenger	passenger	candidate driver	Y
CCNE1	passenger	passenger	passenger	candidate driver	Y
CDK6	passenger	passenger	passenger	candidate driver	Y
CDKN2A	passenger	passenger	passenger	candidate driver	Y
CDKN2B	passenger	passenger	passenger	candidate driver	Y
CNOT3	passenger	passenger	passenger	candidate driver	N
CREBBP	passenger	passenger	passenger	candidate driver	Y
CTCF	passenger	passenger	passenger	candidate driver	Y
CUX1	passenger	passenger	passenger	candidate driver	N
DNMT3A	passenger	passenger	passenger	candidate driver	Y
ECT2L	passenger	passenger	passenger	candidate driver	N
EGFR	passenger	passenger	passenger	candidate driver	Y
ERBB3	passenger	passenger	passenger	candidate driver	Y
ERCC4	passenger	passenger	passenger	candidate driver	Y
FGFR1	passenger	passenger	passenger	candidate driver	Y
FGFR2	passenger	passenger	passenger	candidate driver	Y
FOXP1	passenger	passenger	passenger	candidate driver	N

GNAS	passenger	passenger	passenger	candidate driver	Y
HRAS	passenger	passenger	passenger	candidate driver	Y
IGF1R	passenger	passenger	passenger	candidate driver	Y
KDM6A	passenger	passenger	passenger	candidate driver	Y
MED23	passenger	passenger	passenger	candidate driver	N
MEN1	passenger	passenger	passenger	candidate driver	Y
MLH1	passenger	passenger	passenger	candidate driver	Y
MSH2	passenger	passenger	passenger	candidate driver	Y
MYC	passenger	passenger	passenger	candidate driver	Y
NF2	passenger	passenger	passenger	candidate driver	Y
NOTCH1	passenger	passenger	passenger	candidate driver	Y
NOTCH2	passenger	passenger	passenger	candidate driver	Y
NRAS	passenger	passenger	passenger	candidate driver	Y
PBRM1	passenger	passenger	passenger	candidate driver	Y
PDGFRA	passenger	passenger	passenger	candidate driver	Y
PHF6	passenger	passenger	passenger	candidate driver	N
PMS2	passenger	passenger	passenger	candidate driver	Y
PRDM1	passenger	passenger	passenger	candidate driver	Y
PREX2	passenger	passenger	passenger	candidate driver	N
RHOA	passenger	passenger	passenger	candidate driver	N
SETD2	passenger	passenger	passenger	candidate driver	Y
SF3B1	passenger	passenger	passenger	candidate driver	Y
SMAD4	passenger	passenger	passenger	candidate driver	Y
SMARCA4	passenger	passenger	passenger	candidate driver	Y
SPEN	passenger	passenger	passenger	candidate driver	N
STAG2	passenger	passenger	passenger	candidate driver	Y
STK11	passenger	passenger	passenger	candidate driver	Y
TET2	passenger	passenger	passenger	candidate driver	Y
USP9X	passenger	passenger	passenger	candidate driver	N
XBP1	passenger	passenger	passenger	candidate driver	N
ZFP36L1	passenger	passenger	passenger	candidate driver	N

Supplementary Table 7. Number of samples used in each analysis

Analysis set	Analysis methods	N Metastasis	N Primary	N Dataset	Used data sets_detail
Pan	M, L	4,268	5,217	All	DFCI, MDA, MSK(A), VICC, FMAD, WTSI, INTSERM/TCGA
Distant	M, L	2,328	2,927	Six	MDA, MSK(R), VICC, FMAD, WTSI, INTSERM/TCGA
Local	M, L	119	1,872	Five	DFCI, MDA, MSK(R), VICC, WTSI
Liver	M, L	639	2,786	Four	MSK(R), FMAD, WTSI, INTSERM/TCGA
Lymph nodes	M, L	443	2,921	Five	MSK(R), VICC, FMAD, WTSI, INTSERM/TCGA
Bone	M, L	201	1,870	Two	MSK(R), FMAD
Chest wall/Thorax	M, L	168	1,870	Two	MSK(R), FMAD
Skin	M, L	167	2,786	Four	MSK(R), FMAD, WTSI, INTSERM/TCGA
Lung	M, L	146	1,870	Two	MSK(R), FMAD
Soft tissues	M, L	116	1,870	Two	MSK(R), FMAD
Pleura	M, L	94	1,870	Two	MSK(R), FMAD
Brain	M, L	86	1,870	Two	MSK(R), FMAD
Ovary	M, L	34	1,870	Two	MSK(R), FMAD
Peritoneum	M, L	32	1,870	Two	MSK(R), FMAD
Other distant metastases	M, L	185	2,766	Three	MSK(R), FMAD, INTSERM/TCGA
Histology: IDC	M, L	1,593	3,203	Five	DFCI, MSK(A), VICC, FMAD, WTSI
Histology: ILC	M, L	229	460	Four	DFCI, MSK(A), VICC, FMAD
Receptor status	M, L, F	814	782	Two	MSK(R), WTSI
Between M sites	L	2,160	Not used	Two	MSK(R), FMAD

Abbreviations: MSK(A), MSK data from Razavi *et al.* or GENIE version 8.0; MSK(R), MSK data from Razavi *et al.* M, meta-analysis; L, multivariable logistic regression analysis; F, Fisher's exact test

Supplementary Table 8. MBC enriched altered genes across metastatic site sets compared with PBC samples identified by meta-analysis and multivariable logistic regression analysis

(a) All subtypes

Set	Gene	Collective FDR[a]	Meta-analysis				Heterogeneity test		Multivariable logistic regression analysis			Total number of samples			
			P value	FDR	Test	OR (95%CI)	P value	FDR	P value	FDR	OR (95%CI)	Mmut	Mwt	Pmut	Pwt
Pan	*ESR1*	<0.01	6.30.E-88	1.64.E-85	CMH	7.39 (5.90 to 9.26)	0.04	1	6.92.E-69	1.81.E-66	7.35 (5.91 to 9.23)	554	3714	98	5119
Pan	*SMARCA4*	<0.01	1.77.E-07	4.61.E-05	CMH	2.30 (1.67 to 3.17)	0.94	1	3.31.E-07	8.64.E-05	2.30 (1.68 to 3.19)	110	4158	65	5152
Pan	*NF1*	<0.01	3.80.E-07	9.83.E-05	CMH	1.63 (1.35 to 1.97)	0.06	1	4.59.E-07	1.19.E-04	1.63 (1.35 to 1.98)	272	3996	213	5004
Pan	*FGFR4*	<0.01	5.15.E-06	1.33.E-03	CMH	2.47 (1.66 to 3.69)	0.94	1	9.39.E-06	2.43.E-03	2.46 (1.66 to 3.69)	75	4193	39	5178
Pan	*ARID2*	<0.01	1.55.E-05	3.98.E-03	CMH	1.85 (1.40 to 2.45)	0.56	1	1.96.E-05	5.04.E-03	1.84 (1.39 to 2.43)	131	4137	91	5126
Pan	*PARP1*	<0.05	2.33.E-05	5.97.E-03	CMH	2.85 (1.73 to 4.69)	0.33	1	4.17.E-05	1.07.E-02	2.74 (1.71 to 4.51)	58	4210	27	5190
Pan	*TSC2*	<0.01	2.39.E-05	6.09.E-03	CMH	1.84 (1.38 to 2.47)	0.14	1	3.03.E-05	7.78.E-03	1.88 (1.40 to 2.54)	117	4151	80	5137
Pan	*ATRX*	<0.05	3.58.E-05	9.09.E-03	CMH	1.72 (1.33 to 2.23)	0.17	1	4.18.E-05	1.07.E-02	1.71 (1.33 to 2.22)	149	4119	114	5103
Pan	*ARID1A*	<0.05	5.13.E-05	1.30.E-02	CMH	1.41 (1.19 to 1.66)	0.41	1	5.40.E-05	1.37.E-02	1.40 (1.19 to 1.65)	362	3906	302	4915
Pan	*AURKA*	<0.05	5.45.E-05	1.37.E-02	CMH	3.38 (1.81 to 6.31)	0.35	1	1.26.E-04	3.15.E-02	3.30 (1.83 to 6.27)	39	4229	15	5202
Pan	*STAG2*	<0.05	5.57.E-05	1.40.E-02	CMH	2.01 (1.43 to 2.82)	0.13	1	7.17.E-05	1.81.E-02	1.97 (1.42 to 2.78)	95	4173	62	5155
Pan	*ERBB2*	<0.05	1.07.E-04	2.68.E-02	CMH	1.55 (1.24 to 1.94)	0.08	1	1.17.E-04	2.95.E-02	1.54 (1.24 to 1.92)	197	4071	158	5059
Pan	*ESR1 amp*	<0.05	8.58.E-05	2.17.E-02	CMH	2.92 (1.68 to 5.10)	0.42	1	1.67.E-04	4.24.E-02	2.89 (1.70 to 5.17)	53	1650	17	1657
Distant	*ESR1*	<0.01	4.40.E-52	1.15.E-49	CMH	8.63 (6.23 to 11.95)	0.37	1	5.16.E-40	1.35.E-37	8.68 (6.37 to 12.09)	337	1991	48	2879
Distant	*NF1*	<0.01	6.92.E-06	1.80.E-03	CMH	1.84 (1.41 to 2.41)	0.32	1	8.42.E-06	2.20.E-03	1.83 (1.41 to 2.39)	158	2170	118	2809
Distant	*KEAP1*	<0.05	1.36.E-05	3.53.E-03	CMH	5.49 (2.19 to 13.75)	0.23	1	6.55.E-05	1.69.E-02	7.34 (2.93 to 21.30)	25	2303	6	2921
Distant	*ARID2*	<0.01	1.44.E-05	3.71.E-03	CMH	2.44 (1.61 to 3.69)	0.34	1	2.24.E-05	5.84.E-03	2.44 (1.63 to 3.71)	78	2250	41	2886
Distant	*STAG2*	<0.01	1.65.E-05	4.23.E-03	CMH	2.67 (1.69 to 4.21)	0.53	1	2.59.E-05	6.70.E-03	2.57 (1.67 to 4.03)	68	2260	38	2889
Distant	*FGFR4*	<0.05	6.41.E-05	1.64.E-02	CMH	3.55 (1.83 to 6.89)	0.52	1	1.53.E-04	3.92.E-02	3.54 (1.90 to 7.09)	42	2286	13	2914
Liver	*ESR1*	<0.01	1.68.E-92	4.34.E-90	CMH	18.41 (12.92 to 26.24)	0.06	1	5.45.E-60	1.42.E-57	18.02 (12.85 to 25.74)	169	470	47	2739
Liver	*FGFR4*	<0.05	3.76.E-05	9.65.E-03	CMH	4.54 (2.07 to 9.95)	0.50	1	1.48.E-04	3.77.E-02	4.46 (2.06 to 9.83)	15	624	13	2773
Liver	*STAG2*	<0.05	4.31.E-05	1.10.E-02	CMH	3.23 (1.79 to 5.80)	0.52	1	9.34.E-05	2.39.E-02	3.14 (1.75 to 5.57)	22	617	35	2751
Liver	*ARID2*	<0.01	4.65.E-06	1.20.E-03	CMH	3.18 (1.89 to 5.35)	0.18	1	1.23.E-05	3.18.E-03	3.17 (1.88 to 5.30)	28	611	39	2747
Liver	*NF1*	<0.05	4.56.E-05	1.16.E-02	CMH	2.10 (1.46 to 3.03)	0.15	1	6.29.E-05	1.62.E-02	2.12 (1.46 to 3.05)	48	591	107	2679
Lymph nodes	*ESR1*	<0.01	2.04.E-07	5.28.E-05	CMH	3.45 (2.09 to 5.69)	0.11	1	9.35.E-07	2.43.E-04	3.58 (2.13 to 5.91)	26	417	48	2873
Lymph nodes	*XPO1*	<0.05	3.50.E-05	9.00.E-03	CMH	4.56 (2.11 to 9.88)	0.05	1	1.29.E-04	3.34.E-02	4.47 (2.03 to 9.54)	12	431	23	2898
Bone	*ESR1*	<0.01	4.57.E-27	1.17.E-24	CMH	9.42 (5.74 to 15.45)	0.07	1	1.28.E-19	3.27.E-17	10.03 (6.08 to 16.54)	34	167	38	1832
Bone	*RNF43*	<0.05	1.16.E-06	2.94.E-04	CMH	13.74 (3.67 to 51.43)	0.25	1	1.12.E-04	2.83.E-02	12.21 (3.30 to 45.09)	5	196	5	1865
Bone	*RICTOR*	<0.01	3.67.E-06	9.27.E-04	CMH	5.85 (2.55 to 13.43)	0.33	1	3.36.E-05	8.52.E-03	5.63 (2.41 to 12.57)	10	191	16	1854
Chest wall/ Thorax	*ESR1*	<0.01	1.01E-06	2.55.E-04	CMH	4.33 (2.29 to 8.17)	0.73	1	6.43.E-06	1.63.E-03	4.32 (2.22 to 7.97)	14	154	38	1832
Chest wall/ Thorax	*TP53*	<0.01	3.01E-05	7.58.E-03	CMH	2.06 (1.46 to 2.91)	0.94	1	3.82.E-05	9.65.E-03	2.06 (1.47 to 2.91)	109	59	937	933
Skin	*ESR1*	<0.01	1.62.E-10	4.20.E-08	CMH	5.98 (3.23 to 11.08)	0.92	1	1.16.E-08	3.03.E-06	5.94 (3.14 to 10.75)	16	151	47	2739
Lung	*ESR1*	<0.01	3.54.E-07	9.00.E-05	CMH	4.71 (2.45 to 9.05)	0.29	1	3.36.E-06	8.56.E-04	4.72 (2.37 to 8.85)	13	133	38	1832
Soft tissues	*PARP1*	<0.05	2.95.E-06	7.39.E-04	CMH	7.79 (2.81 to 21.62)	0.52	1	7.17.E-05	1.81.E-02	7.98 (2.68 to 21.69)	6	110	11	1859
Pleura	*ESR1*	<0.01	2.34E-14	5.91E-12	CMH	8.57 (4.46 to 16.49)	0.93	1	1.21.E-10	3.06.E-08	8.57 (4.33 to 16.17)	14	80	38	1832
Brain	*TP53*	<0.01	1.67.E-08	4.24.E-06	CMH	4.27 (2.45 to 7.47)	0.19	1	1.46.E-07	3.72.E-05	4.55 (2.65 to 8.28)	70	16	937	933
Brain	*ROS1*	<0.05	9.25.E-08	2.34.E-05	CMH	20.33 (4.34 to 95.19)	NA	NA	1.31.E-04	3.33.E-02	20.33 (3.86 to 96.51)	3	83	4	1866

Ovary	*CDH1*	<0.01	9.68.E-07	2.43.E-04	CMH	4.76 (2.38 to 9.53)	0.29	1	7.39.E-06	1.87.E-03	4.96 (2.44 to 10.02)	16	18	245	1625
Peritoneum	*ESR1*	<0.01	1.01.E-13	2.55.E-11	CMH	14.15 (5.72 to 35.03)	0.97	1	9.99.E-09	2.53.E-06	14.15 (5.34 to 33.59)	7	25	38	1832
Peritoneum	*CDKN1B*	<0.05	2.04.E-06	5.12.E-04	CMH	11.91 (3.26 to 43.51)	0.73	1	1.79.E-04	4.48.E-02	11.89 (2.65 to 38.69)	3	29	19	1851
Peritoneum	*CDH1*	<0.01	5.55.E-06	1.39.E-03	CMH	4.98 (2.34 to 10.62)	0.55	1	3.30.E-05	8.31.E-03	4.86 (2.24 to 10.12)	12	20	245	1625
Other distant metastases	*ESR1*	<0.01	2.97.E-30	7.67.E-28	CMH	10.66 (6.51 to 17.46)	0.84	1	3.40E-21	8.82E-19	10.70 (6.50 to 17.44)	31	154	47	2719
Other distant metastases	*ATRX*	<0.01	3.29.E-07	8.41.E-05	CMH	4.33 (2.36 to 7.94)	0.08	1	2.37E-06	6.12E-04	4.27 (2.27 to 7.63)	15	170	58	2708
Other distant metastases	*CDKN1B*	<0.05	6.28.E-06	1.60.E-03	CMH	5.17 (2.35 to 11.35)	0.34	1	4.48E-05	1.15E-02	5.08 (2.21 to 10.71)	9	176	29	2737

[a] Collective FDR: FDR is represented by <0.01, <0.05, or <0.1 according to a less significant FDR value from meta-analysis or logistic regression analysis.
[b] Abbreviations: MH, Mantel-Haenszel method; OR, odds ratio; 95% CI, 95% confidence interval; Mmut, MBC samples with the mutated gene; Mwt, MBC samples with the wildtype gene; Pmut, PBC samples with the mutated gene; Pwt, PBC samples with the wildtype gene.

(b) IDC subtypes

Set	Gene	Collective FDR[a]	Meta-analysis				Heterogeneity test		Multivariable logistic regression analysis			Total number of samples			
			**P value**	**FDR**	**Test**	**OR (95%CI)**	**P value**	**FDR**	**P value**	**FDR**	**OR (95%CI)**	**Mmut**	**Mwt**	**Pmut**	**Pwt**
Pan	*ESR1*	<0.01	4.08.E-42	1.06.E-39	CMH	6.38 (4.74 to 8.60)	0.43	1	2.31.E-34	6.05.E-32	6.22 (4.67 to 8.39)	184	1409	65	3138
Pan	*STAG2*	<0.05	1.15.E-04	2.97.E-02	CMH	2.47 (1.54 to 3.97)	0.20	1	1.76.E-04	4.57.E-02	2.44 (1.53 to 3.92)	40	1553	35	3168
Distant	*ESR1*	<0.01	6.01.E-24	1.55.E-21	CMH	6.58 (4.36 to 9.93)	0.39	1	1.21.E-19	3.14.E-17	6.52 (4.40 to 9.92)	122	807	32	1464
Liver	*ESR1*	<0.01	1.12.E-34	2.85.E-32	CMH	12.38 (7.53 to 20.36)	0.31	1	8.89.E-25	2.27.E-22	13.18 (8.12 to 21.78)	53	184	31	1358
Liver	*NF1*	<0.01	1.28.E-05	3.25.E-03	CMH	3.16 (1.83 to 5.43)	0.37	1	2.87.E-05	7.27.E-03	3.16 (1.82 to 5.36)	23	214	47	1342
Liver	*ARID2*	<0.05	1.36.E-05	3.42.E-03	CMH	4.47 (2.18 to 9.17)	0.59	1	4.72.E-05	1.19.E-02	4.25 (2.08 to 8.48)	14	223	23	1366
Liver	*STAG2*	<0.05	3.50.E-05	8.79.E-03	CMH	5.39 (2.21 to 13.11)	0.99	1	1.65.E-04	4.14.E-02	5.38 (2.19 to 12.88)	10	227	13	1376
Bone	*ESR1*	<0.01	3.74.E-19	9.43.E-17	CMH	10.79 (5.58 to 20.86)	0.01	1	1.92.E-13	4.85.E-11	12.84 (6.48 to 25.47)	20	79	31	1358
Chest wall/Thorax	*TP53*	<0.01	2.18E-05	5.46.E-03	CMH	2.80 (1.70 to 4.59)	0.58	1	3.83.E-05	9.62.E-03	2.84 (1.75 to 4.75)	64	24	750	639
Skin	*NF2*	<0.05	9.45.E-06	2.34.E-03	CMH	7.31 (2.55 to 21.0)	0.00	0.11	1.67.E-04	4.18.E-02	8.03 (2.47 to 22.64)	5	60	12	1377
Pleura	*ESR1*	<0.01	1.15E-11	2.86E-09	CMH	11.95 (4.86 to 29.37)	0.58	1	4.81.E-08	1.20.E-05	12.24 (4.74 to 29.24)	8	28	31	1358
Brain	*FGFR4*	<0.01	5.32.E-09	1.32.E-06	CMH	26.10 (5.54 to 123.0)	0.23	1	3.60.E-05	9.01.E-03	22.54 (4.43 to 93.96)	3	26	8	1381
Brain	*FGFR1*	<0.05	1.09.E-08	2.69.E-06	CMH	25.41 (4.56 to 141.55)	0.22	1	1.66.E-04	4.12.E-02	30.07 (3.94 to 163.03)	2	27	9	1380
Other distant metastases	*ESR1*	<0.01	1.02.E-08	2.56.E-06	CMH	8.52 (3.63 to 20.01)	0.18	1	1.09.E-06	2.75.E-04	8.23 (3.32 to 18.48)	8	40	31	1358
Other distant metastases	*KMT2A*	<0.05	1.99.E-05	4.90.E-03	CMH	6.01 (2.37 to 15.27)	0.94	1	1.62.E-04	4.08.E-02	6.014 (2.16 to 14.40)	6	42	36	1353

[a] Collective FDR: FDR is represented by <0.01, <0.05, or <0.1 according to a less significant FDR value from meta-analysis or logistic regression analysis.
[b] Abbreviations: MH, Mantel-Haenszel method; OR, Odds ratio; 95% CI, 95% confidence interval; Mmut, MBC samples with the mutated gene; Mwt, MBC samples with the wildtype gene; Pmut, PBC samples with the mutated gene; Pwt, PBC samples with the wildtype gene

(c) ILC subtype

Set	Gene	Collective FDR[a]	Meta-analysis				Heterogeneity test		Multivariable logistic regression analysis			Total number of samples			
			P value	FDR	Test	OR (95%CI)	P value	FDR	P value	FDR	OR (95%CI)	Mmut	Mwt	Pmut	Pwt
Pan	*ESR1*	<0.01	8.85.E-08	2.18.E-05	CMH	5.76 (2.86 to 11.60)	0.25	1	9.48.E-07	2.34.E-04	5.44 (2.84 to 11.15)	38	260	12	448
Distant	*ESR1*	<0.05	2.90.E-06	6.44.E-04	CMH	8.77 (3.0 to 25.60)	0.66	1	6.34.E-05	1.41.E-02	8.96 (3.40 to 30.89)	27	149	4	214
Other distant metastases	*ESR1*	<0.01	1.18.E-09	2.15.E-07	CMH	19.22 (5.62 to 65.76)	0.59	1	2.35.E-06	4.30.E-04	19.34 (6.01 to 74.75)	10	26	4	201

[a] Collective FDR: FDR is represented by <0.01, <0.05, or <0.1 according to a less significant FDR value from meta-analysis or logistic regression analysis.
[b] Abbreviations: MH, Mantel-Haenszel method; OR, Odds ratio; 95% CI, 95% confidence interval; Mmut, MBC samples with the mutated gene; Mwt, MBC samples with the wildtype gene; Pmut, PBC samples with the mutated gene; Pwt, PBC samples with the wildtype gene

(d) HR+/HER2- subtype

Set	Gene	Collective FDR[a]	Meta-analysis				Heterogeneity test		Multivariable logistic regression analysis			Total number of samples			
			P value	FDR	Test	OR (95%CI)	P value	FDR	P value	FDR	OR (95%CI)	Mmut	Mwt	Pmut	Pwt
Pan	*ESR1*	<0.01	1.18.E-16	2.88.E-14	CMH	7.46 (4.34 to 12.84)	NA	NA	3.96.E-13	9.75.E-11	7.46 (4.46 to 13.31)	96	495	16	602
Distant	*ESR1*	<0.01	5.82.E-17	1.42.E-14	CMH	7.59 (4.41 to 13.06)	NA	NA	2.55.E-13	6.24.E-11	7.59 (4.54 to 13.53)	96	475	16	602
Local	*SMAD4*	<0.05	2.40.E-08	4.82.E-06	CMH	28.62 (4.90 to 167.22)	NA	NA	1.96.E-04	3.96.E-02	28.62 (3.76 to 152.98)	2	18	7	611
Liver	*ESR1*	<0.01	2.02.E-24	4.49.E-22	CMH	13.83 (7.48 to 25.59)	NA	NA	5.82.E-17	1.30.E-14	13.83 (7.61 to 25.26)	40	112	16	602
Liver	*ARID2*	<0.05	3.63.E-07	8.07.E-05	CMH	14.37 (3.86 to 53.47)	0.66	1	7.26.E-05	1.62.E-02	14.06 (4.22 to 63.42)	10	142	3	615
Lymph nodes	*ATR*	<0.05	1.20.E-07	2.59.E-05	CMH	16.44 (4.19 to 64.47)	0.19	1	8.41.E-05	1.82.E-02	18.20 (4.57 to 92.46)	6	74	3	615

[a] Collective FDR: FDR is represented by <0.01, <0.05, or <0.1 according to a less significant FDR value from meta-analysis or logistic regression analysis.
[b] Abbreviations: MH, Mantel-Haenszel method; OR, Odds ratio; 95% CI, 95% confidence interval; Mmut, MBC samples with the mutated gene; Mwt, MBC samples with the wildtype gene; Pmut, PBC samples with the mutated gene; Pwt, PBC samples with the wildtype gene

Supplementary Table 9. Significantly MBC-enriched altered genes across metastatic site sets compared within metastatic sites identified by multivariable logistic regression analysis

(a) All subtypes

Metastatic site	Gene	Multivariable logistic regression analysis			Total number of samples			
		P value	FDR	OR (95%CI)	M site mut	M site wt	Other sites mut	Other sites wt
Liver	*ESR1*	6.25.E-23	1.19.E-21	3.41 (2.67 to 4.35)	163	420	161	1416
Liver	*CDH1*	3.01.E-04	5.12.E-03	0.54 (0.38 to 0.74)	45	538	214	1363
Liver	*TP53*	2.32.E-06	4.18.E-05	0.63 (0.52 to 0.76)	244	339	836	741
Lymph nodes	*ESR1*	1.21.E-07	2.29.E-06	0.30 (0.19 to 0.46)	23	361	301	1475
Lymph nodes	*TP53*	9.27.E-04	1.67.E-02	1.46 (1.17 to 1.83)	225	159	855	921
Bone	*RICTOR*	1.64.E-03	2.95.E-02	3.31 (1.50 to 6.75)	10	191	32	1927
Bone	*TP53*	1.16.E-05	2.20.E-04	0.50 (0.36 to 0.68)	65	136	1015	944
Chest wall/Thorax	*TP53*	1.49.E-05	2.84.E-04	2.09 (1.50 to 2.92)	109	59	971	1021
Lung	*CDH1*	7.54.E-04	1.43.E-02	0.14 (0.03 to 0.37)	3	143	256	1758
Brain	*TP53*	3.94.E-08	7.49.E-07	4.71 (2.78 to 8.47)	70	16	1010	1064
Ovary	*CDH1*	5.96.E-07	1.13.E-05	5.88 (2.90 to 11.79)	16	18	243	1883
Peritoneum	*CDH1*	1.58.E-05	3.00.E-04	5.07 (2.36 to 10.47)	12	20	247	1881
Peritoneum	*TP53*	1.59.E-03	2.86.E-02	0.26 (0.10 to 0.57)	7	25	1073	1055

[a] Collective FDR: FDR is represented by <0.01, <0.05, or <0.1 according to a less significant FDR value from meta-analysis or logistic regression analysis.
[b] Abbreviations: MH, Mantel-Haenszel method; OR, Odds ratio; 95% CI, 95% confidence interval; Mmut, MBC samples with the mutated gene; Mwt, MBC samples with the wildtype gene; Pmut, PBC samples with the mutated gene; Pwt, PBC samples with the wildtype gene

(b) IDC

Metastatic site	Gene	Multivariable logistic regression analysis			Total number of samples			
		P value	FDR	OR (95%CI)	M site mut	M site wt	Other sites mut	Other sites wt
Liver	*ESR1*	6.58.E-06	6.58.E-05	2.49 (1.67 to 3.69)	53	184	69	599
Bone	*TP53*	3.45.E-04	3.45.E-03	0.44 (0.28 to 0.68)	32	67	442	364
Chest wall/Thorax	*TP53*	1.61.E-04	1.61.E-03	2.58 (1.60 to 4.30)	64	24	410	407
Brain	*FGFR4*	3.49.E-03	3.49.E-02	7.19 (1.57 to 24.23)	3	26	13	863

(c) HR+/HER2-

Metastatic site	Gene	Multivariable logistic regression analysis			Total number of samples			
		P value	FDR	OR (95%CI)	M site mut	M site wt	Other sites mut	Other sites wt
Liver	*ESR1*	1.20.E-04	3.59.E-04	2.46 (1.55 to 3.89)	40	107	56	369

Supplementary Table 10. Patient samples biopsied from at least two metastatic sites used in analysis of metastasis site-specific analysis

Patient ID	Cohort	Liver	LN	Bone	Chest wall/ Thorax	Skin	Lung	Soft tissue	Pleura	Brain	Ovary	Peritoneum
P-0000066	MSK(R)	0	0	0	1	0	1	0	0	0	0	0
P-0000081	MSK(R)	1	0	1	0	0	0	0	0	0	0	0
P-0000104	MSK(R)	1	1	0	0	0	0	0	0	0	0	0
P-0000167	MSK(R)	0	1	1	0	0	0	0	0	0	0	0
P-0000234	MSK(R)	1	0	0	1	0	0	0	0	0	0	0
P-0000247	MSK(R)	1	1	0	0	0	0	0	0	0	0	0
P-0000337	MSK(R)	1	0	1	0	0	0	0	0	0	0	0
P-0000422	MSK(R)	1	0	0	0	0	0	0	0	0	1	0
P-0000584	MSK(R)	0	1	0	0	0	0	0	0	0	1	0
P-0000690	MSK(R)	1	0	1	0	0	0	1	0	0	0	0
P-0000984	MSK(R)	0	1	0	1	0	0	0	0	0	0	0
P-0001338	MSK(R)	0	1	0	0	0	0	1	0	0	0	0
P-0001396	MSK(R)	0	0	0	1	1	0	0	0	0	0	0
P-0001484	MSK(R)	0	1	0	1	0	0	0	0	0	0	0
P-0001505	MSK(R)	1	0	0	0	0	1	0	0	0	0	0
P-0002088	MSK(R)	0	0	0	0	0	0	1	0	0	0	1
P-0002281	MSK(R)	0	0	1	1	0	0	0	0	0	0	0
P-0002562	MSK(R)	0	0	0	1	0	0	1	0	0	0	0
P-0002995	MSK(R)	0	1	0	1	0	0	0	0	0	0	0
P-0003198	MSK(R)	0	0	0	1	1	0	0	0	0	0	0
P-0003223	MSK(R)	1	0	1	0	0	0	0	0	0	0	0
P-0003273	MSK(R)	0	1	1	0	0	0	0	0	0	0	0
P-0003866	MSK(R)	0	1	0	0	0	0	0	1	0	0	0
P-0003903	MSK(R)	1	0	1	0	0	0	0	0	0	0	0
P-0003933	MSK(R)	1	1	0	0	0	0	0	0	0	0	0
P-0003950	MSK(R)	1	1	0	0	0	0	0	0	0	0	0
P-0003958	MSK(R)	0	0	1	0	0	0	0	0	0	1	0
P-0004728	MSK(R)	1	0	1	0	0	0	0	0	0	0	0
P-0004835	MSK(R)	1	1	0	0	0	0	0	0	0	0	0
P-0004909	MSK(R)	0	1	0	1	0	0	0	0	0	0	0
P-0005131	MSK(R)	0	0	0	1	0	1	0	0	0	0	0
P-0005268	MSK(R)	1	0	1	0	0	0	0	0	0	0	0
P-0006161	MSK(R)	1	0	1	0	0	0	0	0	0	0	0
P-0006227	MSK(R)	0	0	0	0	0	0	0	1	0	1	0
P-0006376	MSK(R)	1	1	0	0	0	0	0	0	0	0	0
P-0006544	MSK(R)	1	1	0	0	0	0	0	0	0	0	0
P-0006910	MSK(R)	0	0	0	1	1	0	0	0	0	0	0
P-0007009	MSK(R)	0	0	1	0	0	0	1	0	0	0	0
P-0007129	MSK(R)	0	1	0	0	0	0	0	0	0	0	1
P-0008179	MSK(R)	1	0	0	0	0	0	0	0	0	1	0
P-0008334	MSK(R)	0	1	1	0	0	0	0	0	0	0	0
P-0009406	MSK(R)	0	1	0	0	0	1	0	0	0	0	0
P-0009874	MSK(R)	0	0	0	0	0	0	0	1	0	1	0
P-0012855	MSK(R)	1	0	0	0	0	0	0	1	0	0	0
P-0014628	MSK(R)	0	1	0	1	0	0	0	0	0	0	0
P-0014703	MSK(R)	1	0	0	0	0	1	0	0	0	0	0
PD8709	WTSI	0	1	0	0	1	0	0	0	0	0	0
PD8715	WTSI	0	1	0	0	1	0	0	0	0	0	0

Supplementary Table 11. Scoring criteria of variants to assess functional impact

	**Functional score**			
**Annotator**	**3**	**2**	**1**	**0**
**Mutation Assessor**	high	medium	low	neutral
**Polyphen2**	deleterious	-	low	tolerated
**SIFT**	probably_damaging	-	possibly_damaging	benign
**Literature**	In breast cancer	In >1 other cancer types	1 other cancer types	none

Supplementary Table 12. Functional annotation of variants for driver mutation analysis

Set	Gene	Chr	Start	End	Hotspot Region	CGC	OG/TSG	MA	PPh2	SIFT	PMID score	PMID: Breast cancer	PMID: Other cancer types	Func Score	Candidate driver?	Driver likelihood[a]
Pan, Distant, Liver, Lymph nodes, Bone, Chest/Thorax, Skin, Other distant metastases	*ESR1*	6	152332832	152332832	E380Q	Y	OG	1	3	3	Not Required	-	-	7	Y	1
Pan, Distant	*ESR1*	6	152419920	152419920	L536R	Y	OG	2	3	1	Not Required	-	-	6	Y	1
Pan, Distant, Liver	*ESR1*	6	152419920	152419920	L536H	Y	OG	2	3	3	Not Required	-	-	8	Y	1
Pan, Distant, Liver	*ESR1*	6	152419920	152419920	L536P	Y	OG	1	3	3	Not Required	-	-	7	Y	1
Pan, Distant, Liver, Lymph nodes, Bone, Lung, Peritoneum	*ESR1*	6	152419922	152419922	Y537N	Y	OG	1	3	3	Not Required	-	-	7	Y	1
Pan, Distant, Liver, Lymph nodes, Bone, Lung, Pleura, Peritoneum, Other distant metastases	*ESR1*	6	152419923	152419923	Y537S	Y	OG	2	3	3	Not Required	-	-	8	Y	1
Pan, Distant, Liver, Lymph nodes, Skin, Other distant metastases	*ESR1*	6	152419923	152419923	Y537C	Y	OG	1	3	3	Not Required	-	-	7	Y	1
Pan, Distant, Liver, Lymph nodes, Bone, Chest/Thorax, Skin, Lung, Pleura, Other distant metastases	*ESR1*	6	152419926	152419926	D538G	Y	OG	2	3	1	Not Required	-	-	6	Y	1
Pan, Distant, Liver	*FGFR4*	5	176522416	176522416	N535K	Y	OG	0	3	3	Not Required	-	-	6	Y	NA
Pan, Distant, Liver	*FGFR4*	5	176522551	176522551	V550L	Y	OG	0	3	3	Not Required	-	-	6	Y	NA
Pan, Distant	*FGFR4*	5	176522551	176522551	V550M	Y	OG	0	3	3	Not Required	-	-	6	Y	NA
Pan, Distant, Liver	*STAG2*	X	NA	NA	Truncated	Y	TSG	NA	NA	NA	5	28430577	24484537;24121791; 21852505;28691904	5	Y	0.53
Pan, Distant, Liver	*NF1*	17	NA	NA	Truncated	Y	TSG	NA	NA	NA	5	22851646	15937108;24576830; 8563751	5	Y	0.94
Pan	*SMARCA4*	19	NA	NA	Truncated	Y	TSG	NA	NA	NA	5	17637742	25370573;19789351	5	Y	0.46
Pan	*TSC2*	16	NA	NA	Truncated	Y	TSG	NA	NA	NA	5	16537497	16388022;25724664	5	Y	NA
Pan	*ARID1A*	1	NA	NA	Truncated	Y	TSG	NA	NA	NA	5	31913353	22484628;31217031	5	Y	0.98
Distant	*KEAP1*	19	NA	NA	Truncated	Y	TSG	NA	NA	NA	5	17822677	20124447;18316592; 20124447	5	Y	0.45
Brain	*TP53*	17	7577121	7577121	R273C	Y	OG	2	3	3	Not Required	-	-	8	Y	1
Chest/Thorax	*TP53*	17	7577538	7577538	R248Q	Y	OG	2	3	3	Not Required	-	-	8	Y	1
Brain	*TP53*	17	7577539	7577539	R248W	Y	OG	2	3	3	Not Required	-	-	8	Y	1
Chest/Thorax, Brain	*TP53*	17	7577547	7577547	G245D	Y	OG	2	3	3	Not Required	-	-	8	Y	1
Chest/Thorax	*TP53*	17	7577570	7577570	M237I	Y	OG	2	3	3	Not Required	-	-	8	Y	1
Chest/Thorax	*TP53*	17	7577574	7577574	Y236C	Y	OG	2	3	3	Not Required	-	-	8	Y	NA
Brain	*TP53*	17	7578535	7578535	K132R	Y	OG	2	3	3	5	-	-	13	Y	1
Chest/Thorax, Brain	*TP53*	17	NA	NA	Truncated	Y	TSG	NA	NA	NA	5	17097565;17626182	12154352;11507071	5	Y	0.99
Ovary, Peritoneum	*CDH1*	16	NA	NA	Truncated	Y	TSG	NA	NA	NA	5	19350629;17097565	26901067;24211838	5	Y	0.99
Peritoneum, Other metastases	*CDKN1B*	12	NA	NA	Truncated	Y	TSG	NA	NA	NA	5	28377607	30992462;30992462	5	Y	0.93

Abbreviations: Chr, chromosome; CGC, Cancer Gene Census; OG, oncogene; TSG, tumor suppressor gene; MA, Mutation Assessor; PPh2, polyphen2; Func score, functional score related to Supplementary Table 10.

[a] For OG, driver likelihood was from the original value described in a published paper (PMID:31645765) and for TSG, mean of driver likelihood on potential truncating variants (frame-shift indel, splice, and nonsense) specified in the paper was estimated. NA means the gene was not involved in the analysis of breast cancer in the study.

Supplementary Table 13. Result of meta-analysis and logistic regression analysis for candidate driver mutations by comparing MBC with PBC

Set	Gene	Amino acid Change	Collective FDR[a]	Meta-analysis				Heterogeneity test		Multivariable logistic regression analysis			Total number of samples			
				P value	FDR	Test	OR (95%CI)	P value	FDR	P value	FDR	OR (95%CI)	Mmut	Mwt	Pmut	Pwt
Pan	*ESR1*	D538G	<0.01	7.52.E-42	1.20.E-40	CMH	16.93 (9.67 to 29.62)	0.48	1	8.67.E-24	1.65.E-22	18.34 (10.84 to 33.99)	202	4066	13	5204
Pan	*ESR1*	Y537S	<0.01	9.02.E-27	1.35.E-25	CMH	20.97 (9.61 to 45.76)	0.23	1	1.16.E-14	2.09.E-13	20.58 (10.29 to 48.90)	125	4143	7	5210
Pan	*ESR1*	E380Q	<0.01	2.18.E-09	2.84.E-08	CMH	5.74 (3.03 to 10.85)	0.16	1	6.18.E-08	1.05.E-06	5.65 (3.12 to 11.03)	53	4215	13	5204
Pan	*ESR1*	Y537N	<0.01	1.83.E-10	2.57.E-09	CMH	16.34 (5.09 to 52.46)	0.94	1	2.14.E-06	3.42.E-05	17.07 (6.21 to 70.58)	50	4218	3	5214
Pan	*ESR1*	Y537C	<0.01	8.15.E-06	9.78.E-05	CMH	7.41 (2.60 to 21.14)	0.72	1	1.37.E-04	1.78.E-03	7.90 (3.05 to 26.98)	28	4240	4	5213
Pan	*NF1*	Trunc	<0.01	8.12.E-05	7.96.E-04	CMH	1.62 (1.27 to 2.06)	0.09	1	9.15.E-05	1.28.E-03	1.62 (1.27 to 2.06)	166	4102	129	5088
Pan	*ARID1A*	Trunc	<0.01	9.59.E-06	1.05.E-04	CMH	1.58 (1.29 to 1.94)	0.71	1	1.09.E-05	1.64.E-04	1.58 (1.29 to 1.93)	237	4031	183	5034
Distant	*ESR1*	E380Q	<0.01	1.76E-06	2.29E-05	CMH	11.81 (3.38 to 41.30)	0.33	1	3.85E-05	5.39E-04	10.44 (3.81 to 37.33)	27	2170	2	2009
Distant	*ESR1*	Y537C	<0.05	4.19.E-04	4.19.E-03	CMH	8.47 (2.01 to 35.72)	0.22	1	3.42.E-03	3.42.E-02	8.79 (2.56 to 55.12)	20	2177	2	2009
Distant	*ESR1*	Y537N	<0.01	2.05E-06	2.46E-05	CMH	14.34 (3.40 to 60.49)	0.81	1	2.92.E-04	3.50.E-03	14.04 (4.26 to 86.70)	33	2164	2	2009
Distant	*ESR1*	Y537S	<0.01	3.54E-16	4.96E-15	CMH	37.64 (9.14 to 155.02)	0.15	1	3.62E-07	5.43E-06	39.83 (12.31 to 244.68)	75	2122	2	2009
Distant	*ESR1*	D538G	<0.01	2.72E-24	4.09E-23	CMH	19.47 (8.66 to 43.78)	0.38	1	1.97E-13	3.15E-12	23.16 (10.92 to 60.04)	119	2078	6	2005
Distant	*ARID1A*	Trunc	<0.01	2.07.E-04	2.27.E-03	CMH	1.71 (1.29 to 2.28)	0.87	1	2.31.E-04	3.00.E-03	1.70 (1.28 to 2.26)	129	2068	71	1940
Distant	*NF1*	Trunc	<0.05	4.35.E-03	3.48.E-02	CMH	1.64 (1.17 to 2.30)	0.45	1	4.60.E-03	4.14.E-02	1.63 (1.16 to 2.28)	86	2111	50	1961
Distant	*STAG2*	Trunc	<0.05	6.21.E-04	5.59.E-03	CMH	4.20 (1.72 to 10.24)	0.98	1	1.10.E-03	1.21.E-02	4.16 (1.83 to 10.31)	21	2176	5	2006
Liver	*ESR1*	E380Q	<0.01	2.23.E-10	1.78.E-09	CMH	21.67 (5.78 to 81.18)	0.161	1	1.05.E-06	1.05.E-05	19.88 (6.52 to 75.66)	14	625	4	2782
Liver	*ESR1*	Y537N	<0.01	2.50E-13	2.25E-12	CMH	33.54 (7.68 to 146.52)	0.559	1	2.86.E-06	2.57.E-05	32.48 (9.44 to 203.98)	20	619	2	2784
Liver	*ESR1*	Y537S	<0.01	2.24.E-27	2.24.E-26	CMH	74.18 (17.13 to 321.23)	0.192	1	5.21.E-09	5.73.E-08	72.14 (21.74 to 447.63)	38	601	2	2784
Liver	*ESR1*	D538G	<0.01	1.20.E-48	1.32.E-47	CMH	43.89 (19.08 to 100.98)	0.472	1	2.51.E-19	3.02.E-18	49.12 (22.82 to 128.38)	73	566	6	2780
Liver	*STAG2*	Trunc	<0.1	7.07.E-03	2.98.E-02	CMH	4.48 (1.41 to 14.26)	0.291	1	1.19.E-02	7.15.E-02	4.06 (1.34 to 12.33)	7	632	8	2778
Liver	*NF1*	Trunc	<0.01	9.29.E-04	5.58.E-03	CMH	2.12 (1.35 to 3.34)	0.436	1	1.17.E-03	9.34.E-03	2.12 (1.33 to 3.31)	31	608	68	2718
Lymph nodes	*ESR1*	Y537C	<0.01	1.40.E-03	4.51.E-03	CMH	8.48 (1.65 to 43.61)	0.14	0.41	8.77.E-03	3.31.E-02	9.91 (1.90 to 71.40)	4	433	2	2900
Lymph nodes	*ESR1*	Y537S	<0.01	2.78.E-05	1.39.E-04	CMH	16.72 (2.99 to 93.53)	0.02	0.07	1.44.E-03	8.62.E-03	17.90 (3.45 to 140.73)	5	438	2	2919
Lymph nodes	*ESR1*	D538G	<0.01	1.13.E-03	4.51.E-03	CMH	4.95 (1.63 to 15.01)	0.01	0.07	3.62.E-03	1.81.E-02	5.74 (1.73 to 19.29)	6	431	6	2896
Bone	*ESR1*	E380Q	<0.05	2.40.E-03	2.40.E-03	CMH	9.76 (1.61 to 59.01)	NA	NA	1.31.E-02	1.31.E-02	9.76 (1.60 to 74.66)	3	198	2	1868
Bone	*ESR1*	Y537N	<0.01	1.08.E-04	2.16.E-04	CMH	13.54 (2.26 to 80.95)	0.426	0.852	3.21.E-03	6.41.E-03	15.58 (2.49 to 121.51)	3	198	2	1868
Bone	*ESR1*	Y537S	<0.01	1.09.E-19	4.36.E-19	CMH	37.95 (9.37 to 153.63)	0.045	0.134	1.74.E-07	8.72.E-07	59.05 (15.40 to 387.46)	11	190	2	1868
Bone	*ESR1*	D538G	<0.01	1.05.E-11	3.14.E-11	CMH	17.00 (5.77 to 50.12)	0.469	0.852	1.99.E-07	8.72.E-07	15.80 (5.70 to 47.59)	10	191	6	1864
Chest/Thorax	*ESR1*	D538G	<0.01	1.35.E-07	9.47.E-07	CMH	11.66 (3.70 to 36.77)	0.679	1	2.79.E-05	2.24.E-04	11.55 (3.57 to 37.36)	6	162	6	1864
Chest/Thorax	*TP53*	Y236C	<0.05	7.50.E-07	4.50.E-06	CMH	37.08 (3.71 to 370.79)	0.540	1	2.08.E-03	1.45.E-02	35.47 (4.50 to 720.41)	3	165	1	1869
Chest/Thorax	*ESR1*	E380Q	<0.05	3.74.E-05	1.87.E-04	CMH	15.22 (2.61 to 88.85)	0.112	0.787	2.28.E-03	1.45.E-02	16.45 (2.70 to 125.77)	3	165	2	1868
Skin	*ESR1*	E380Q	<0.01	2.15.E-04	4.29.E-04	CMH	16.66 (2.55 to 109.06)	0.476	0.952	3.75.E-03	7.50.E-03	13.63 (1.79 to 74.42)	2	165	4	2782
Skin	*ESR1*	Y537C	<0.01	1.96.E-08	5.88.E-08	CMH	19.94 (4.06 to 97.99)	0.051	0.153	1.58.E-04	4.73.E-04	31.20 (5.62 to 240.81)	4	163	2	2784
Lung	*ESR1*	Y537N	<0.05	9.79.E-04	1.91.E-03	CMH	12.96 (1.81 to 92.56)	0.248	0.497	1.07.E-02	3.19.E-02	12.96	2	144	2	1868
Lung	*ESR1*	Y537S	<0.05	9.53.E-04	1.91.E-03	CMH	12.92 (1.81 to 92.29)	0.046	0.137	1.06.E-02	3.19.E-02	13.0 (1.55 to 109.01)	2	144	2	1868
Lung	*ESR1*	D538G	<0.01	6.22.E-05	1.86.E-04	CMH	8.74 (2.44 to 31.29)	0.586	0.586	8.68.E-04	3.47.E-03	8.75 (2.22 to 30.98)	4	142	6	1864
Pleura	*ESR1*	Y537S	<0.01	1.54.E-09	1.54.E-09	CMH	35.59 (5.65 to 224.29)	0.408	0.816	1.34.E-04	2.68.E-04	34.33 (5.56 to 265.86)	3	91	2	1868
Pleura	*ESR1*	D538G	<0.01	4.47.E-13	8.94.E-13	CMH	20.94 (6.59 to 66.53)	0.579	0.816	2.61.E-07	7.83.E-07	20.71 (6.35 to 67.58)	6	88	6	1864
Brain	*TP53*	G245D	<0.05	9.46.E-04	2.84.E-03	CMH	10.41 (1.86 to 58.09)	NA	NA	7.59.E-03	3.04.E-02	10.41 (1.42 to 54.56)	2	84	4	1866
Brain	*TP53*	K132R	<0.05	1.45.E-04	7.25.E-04	CMH	14.60 (2.38 to 89.58)	0.397	1.000	3.87.E-03	1.93.E-02	14.31 (1.86 to 87.67)	2	84	3	1867
Brain	*TP53*	Trunc	<0.01	1.95.E-04	7.79.E-04	CMH	2.29 (1.46 to 3.58)	0.513	1.000	2.75.E-04	1.65.E-03	2.30 (1.46 to 3.60)	34	52	409	1461
Ovary	*CDH1*	Trunc	<0.01	1.22.E-05	1.22.E-05	CMH	4.24 (2.09 to 8.60)	0.433	0.433	5.03.E-05	1.01.E-04	4.36 (2.10 to 8.81)	14	20	221	1649
Peritoneum	*ESR1*	Y537S	<0.01	2.29.E-42	6.87.E-42	CMH	252.61 (33.49 to 1905.62)	0.38	0.86	1.64.E-08	6.56.E-08	202.17 (34.71 to 1647.75)	4	28	2	1868
Peritoneum	*ESR1*	Y537N	<0.01	7.39.E-15	1.48.E-14	CMH	66.84 (8.79 to 508.55)	0.69	0.86	4.09.E-05	1.23.E-04	71.67 (8.10 to 642.91)	2	30	2	1868
Peritoneum	*CDH1*	Trunc	<0.01	1.20.E-04	1.20.E-04	CMH	4.30 (1.94 to 9.53)	0.29	0.86	3.54.E-04	7.08.E-04	4.14 (1.82 to 8.81)	10	22	221	1649

Peritoneum	*CDKN1B*	Trunc	<0.01	5.97.E-04	5.97.E-04	CMH	9.16 (1.97 to 42.52)	0.85	1.00	4.66.E-03	9.32.E-03	9.18 (1.39 to 35.41)	2	30	16	1854
Other distant metastases	*ESR1*	D538G	<0.01	4.93.E-28	2.47.E-27	CMH	30.78 (11.90 to 79.63)	0.39	1	1.07.E-12	6.42.E-12	34.04 (13.50 to 97.50)	15	171	6	2766
Other distant metastases	*ESR1*	Y537S	<0.01	2.36.E-20	9.44.E-20	CMH	66.38 (13.25 to 332.57)	0.20	0.99	2.48E-07	1.24E-06	58.71 (14.87 to 388.99)	9	177	2	2770
Other distant metastases	*CDKN1B*	Trunc	<0.01	1.42.E-05	4.27.E-05	CMH	5.30 (2.30 to 12.20)	0.49	1	9.27E-05	3.71E-04	5.21 (2.14 to 8.42)	8	178	26	2746
Other distant metastases	*ESR1*	E380Q	<0.01	4.37.E-05	6.68.E-05	CMH	15.30 (2.89 to 80.87)	0.29	1	1.33.E-03	4.00.E-03	12.81 (2.41 to 61.96)	3	183	4	2768
Other distant metastases	*ESR1*	Y537C	<0.01	3.34.E-05	6.68.E-05	CMH	15.77 (2.67 to 93.01)	0.30	1	2.48E-07	1.24E-06	58.71 (14.87 to 388.99)	3	183	2	2770

[a] Collective FDR: FDR is represented by <0.01, <0.05, or <0.1 according to a less significant FDR value from meta-analysis or logistic regression analysis.
[b] Abbreviations: MH, Mantel-Haenszel method; REML, restricted maximum likelihood method; OR, Odds ratio; 95% CI, 95% confidence interval; Mmut, MBC samples with the mutated gene; Mwt, MBC samples with the wildtype gene; Pmut, PBC samples with the mutated gene; Pwt, PBC samples with the wildtype gene; Trunc, truncating mutations

Supplementary Table 14. Significantly MBC-enriched driver mutations across metastatic site sets compared within metastatic sites identified by multivariable logistic regression analysis

Metastatic site	Gene	Multivariable logistic regression analysis			Total number of samples			
		P value	FDR	OR (95%CI)	M site mut	M site wt	Other sites mut	Other sites wt
Liver	*ESR1_Y537N*	3.49.E-05	8.72.E-04	4.60 (2.26 to 9.75)	20	563	12	1565
Liver	*ESR1_Y537S*	2.00.E-05	5.21.E-04	2.74 (1.72 to 4.36)	37	546	38	1539
Liver	*ESR1_D538G*	7.26.E-14	1.96.E-12	4.24 (2.91 to 6.23)	70	513	49	1528
Lymph nodes	*ESR1_D538G*	2.86.E-04	7.71.E-03	0.19 (0.07 to 0.42)	5	379	114	1662
Brain	*TP53_Trunc*	7.54.E-05	2.04.E-03	2.47 (1.57 to 3.84)	34	52	437	1637
Ovary	*CDH1_Trunc*	2.38.E-06	6.42.E-05	5.50 (2.66 to 11.08)	14	20	205	1921
Peritoneum	*CDH1_Trunc*	9.01.E-05	2.43.E-03	4.68 (2.07 to 9.87)	10	22	209	1919

[a] Collective FDR: FDR is represented by <0.01, <0.05, or <0.1 according to a less significant FDR value from meta-analysis or logistic regression analysis.
[b] Abbreviations: MH, Mantel-Haenszel method; OR, Odds ratio; 95% CI, 95% confidence interval; Mmut, MBC samples with the mutated gene; Mwt, MBC samples with the wildtype gene; Pmut, PBC samples with the mutated gene; Pwt, PBC samples with the wildtype gene
